# EGCG-Enhanced Ferrous-Activated Peroxydisulfate Processes for *Shigella flexneri* Biofilm Inactivation: Efficacy, Underlying Mechanisms, and Practical Implications

**DOI:** 10.3390/microorganisms14081717

**Published:** 2026-08-05

**Authors:** Xuan Liu, Yu Chen, Zheng Ji, Yeyue Zhang, Yini Zhang, Yuru Wang, Minrui Li

**Affiliations:** 1 School of Geography and Tourism, Shaanxi Normal University, Xi’an 710119, China; liuxuan2023@snnu.edu.cn (X.L.); 20242419@snnu.edu.cn (Y.C.); zhangyeyue0223@163.com (Y.Z.); wangyuru@snnu.edu.cn (Y.W.); liminrui503@126.com (M.L.); 2 International Joint Research Centre of Shaanxi Province for Pollutants Exposure and Eco-Environmental Health, Xi’an 710119, China; 3 School of Environment, Northeast Normal University, Changchun 130117, China; zoeyyy97@163.com

**Keywords:** epigallocatechin gallate, *Shigella flexneri* biofilm, ferrous-activated peroxydisulfate, advanced oxidation process, disinfection

## Abstract

*Shigella flexneri* (*S. flexneri*), as a common foodborne and waterborne pathogen, poses a serious health threat, with its biofilm formation significantly increasing disinfection difficulty. Ferrous-activated peroxydisulfate (Fe^2+^/PDS) is effective for pollutant removal but limited by unstable Fe^2+^. To address this issue, the present study developed an epigallocatechin gallate (EGCG)-enhanced Fe^2+^/PDS system for inactivating *S. flexneri* biofilms. The biofilm biomass and viability were assessed via crystal violet staining and CLSM. The results showed that comparable biomass reduction and inactivation was achieved with the EGCG-enhanced system at much lower iron loading. Mechanistic analysis revealed that compared with the original system, the EGCG-enhanced system maintained a higher effective Fe^2+^ concentration, likely contributing to a more stable Fe^2+^/Fe^3+^redox cycle. High-valent Fe species and multiple reactive oxygen species were also observed in the enhanced system which might contribute to the biomass reduction and inactivation. Compared with the Fe^2+^/PDS system, the EGCG-enhanced system developed in this work achieved a broader pH adaptability and stronger anti-interference capability in tap water, and exhibited lower biological toxicity. These findings provide new perspectives for green disinfection technologies and agricultural waste utilization.

## 1. Introduction

*Shigella flexneri* (*S. flexneri*), commonly transmitted through contaminated food or water, is the leading cause of endemic diarrhea in low- and middle-income countries [1,2]. Notably, 78.5% of *S. flexneri* strains exhibit multi-drug resistance [2,3]. The resulting health crises and economic burdens, coupled with ever-increasing medical costs, might pose a tough challenge to society.

Bacterial biofilms, as multicellular consortia, possess quite stable structures. In particular, the extracellular polymeric substances (EPSs), in the outer layer of biofilm, could act as robust barriers, shielding the embedded microorganisms and thereby conferring enhanced resistance to diverse environmental stresses [4]. It was estimated that over 80% of microbial infections in humans might be related to biofilms [5]. When biofilms are formed on food processing machines and implanted medical devices, subsequent cross-contaminations and infections might occur and even lead to death [6]. Bacterial biofilm in diverse water supply systems (for food processing and production, livestock breeding, drinking water, etc.) could accumulate pollutants, facilitate the formation and transfer of antibiotic resistance, etc. Meanwhile, microorganisms and their metabolins could act as significant precursors of disinfection byproducts [7,8,9]. Ultimately, all these could pose a significant threat to food safety and human health [4,10]. Moreover, biofilm could also lead to the corrosion of water supply pipes and other devices resulting in the increase in color, odor, and turbidity, and a decrease in the qualities of related products [11].

Traditional biofilm removal methods, such as mechanical scraping and chemical disinfection, often present poor removal effects or potential adverse contamination of the environment. Advanced oxidation processes (AOPs) have shown distinct advantages in organic pollutant removal. A free radical chain reaction generated in AOPs was suggested as one of the most active and effective oxidants to inactivate microorganisms [12]. Among them, ferrous-activated peroxydisulfate (Fe^2+^/PDS) has become a better choice because of its good catalytic effect and more cost-effective performance. Activated PDS could produce both hydroxyl radicals (^•^OH) and sulfate radicals (SO_4_^•−^) to oxidize pollutants. However, Fe (II), which acts as the activator in Fe^2+^/PDS, could be oxidized into Fe (III) easily and would form precipitation when pH is near neutral or alkaline, which might affect the generation of free radicals [13]. Thus, modification of the Fe^2+^/PDS system is still of great necessity.

**Figure 1:**
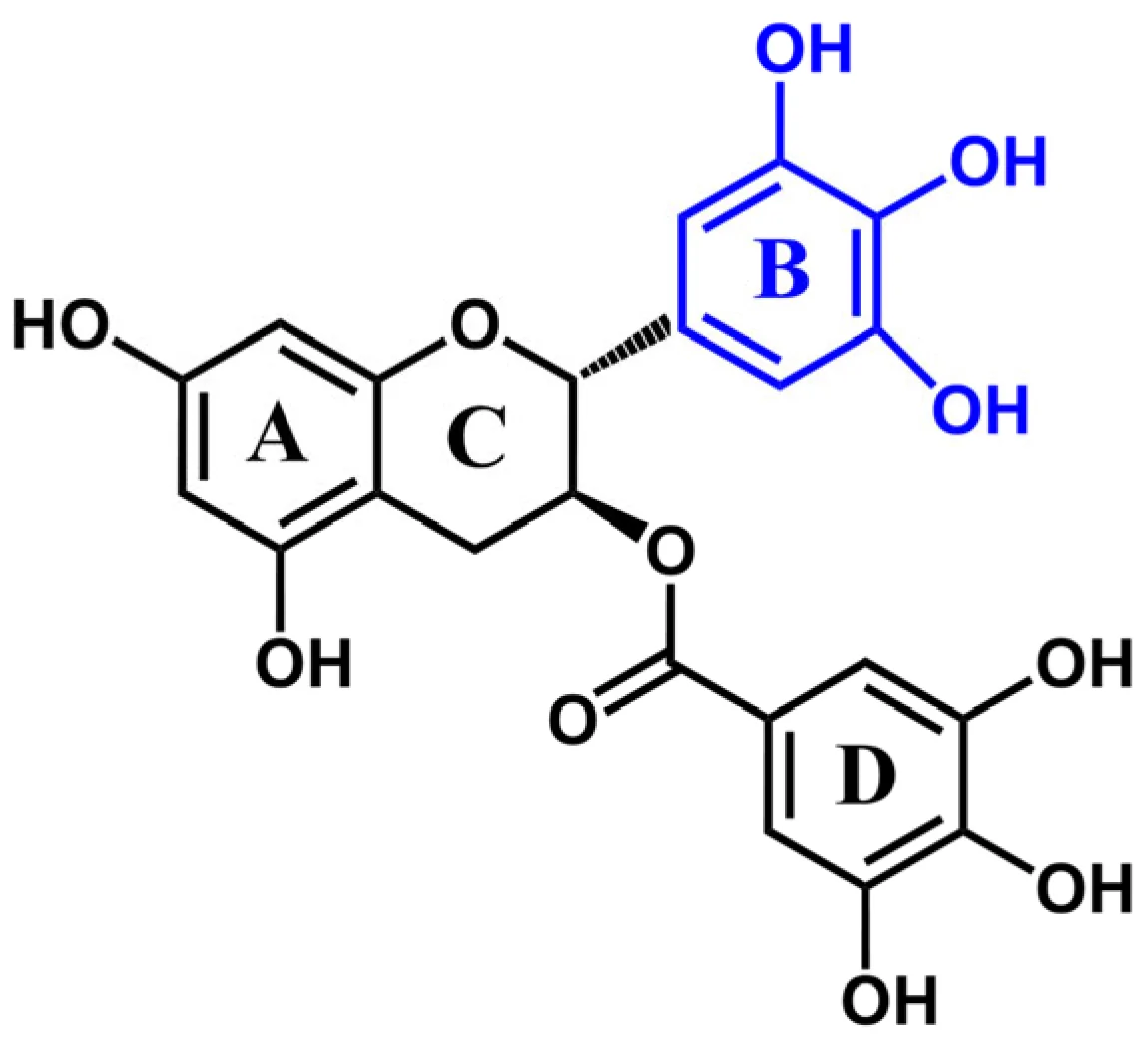
Structural formula of EGCG. (A–D: ring designations; the B ring is highlighted in blue).

As a big country for tea production and consumption, China lacks deep and comprehensive utilization and application of tea products, which might result in a huge waste of resources. Epigallocatechin gallate (EGCG, shown in Figure 1), which is extracted from tea leaves, exhibits stronger antibacterial effects compared to other tea polyphenols [14]. The better antibacterial property of EGCG might lie in the higher concentration of active phenolic hydroxyl groups. Researchers indicated that EGCG could directly bind with peptidoglycans, disrupting cell walls and interfering with their biosynthesis [15]. Additionally, EGCG could also be oxidized into EGCG polymers and quinone substances when catalyzed by metal ions. This process, accompanied by the generation of reactive oxygen species (ROS), would contribute to a better antibacterial effect [16]. There are several investigations focusing on the removal efficiency of certain chemicals by tea polyphenol-enhanced Fe^2+^/PDS systems [17,18]. However, the anti-biofilm activity of EGCG-enhanced Fe^2+^/PDS systems against *S. flexneri* has rarely been reported in any depth. Meanwhile, the existence of other reactive species and the variation in toxicity have not been extensively investigated.

EGCG was applied here as a booster of the Fe^2+^/PDS system. The aim of the study was to evaluate the efficacy of an EGCG-enhanced Fe^2+^/PDS system on mature *S. flexneri* biofilms. The physicochemical and microbiological characterization was investigated, and a hypothesis of the reaction mechanism was proposed. The acute toxicity was also assessed. Further application using tap water was conducted as well. This work contributes to the development of a more environmentally friendly and efficient strategy for addressing biofilm contamination issues and gives new insight for tea product applications.

## 2. Materials and Methods

### 2.1. Materials

*S. flexneri* (ATCC 12022) was purchased from Hope Bio-Technology Co., Ltd. (Qingdao, China). *Vibrio fischeri* (*V. fischeri*, ATCC 25918) was purchased from the China Center of Industrial Culture Collection. EGCG (purity ≥ 98%) was purchased from Shanghai Yuanye Bio-Technology Co., Ltd. (Shanghai, China). Other details of chemicals and kits used in the study are described in Text S1.

### 2.2. S. flexneri Biofilm Cultivation and AOP Treatment

*S. flexneri* was activated according to previous research [7]. The activated bacterial suspension was diluted with 1/2 LB broth to make the initial concentration in each reaction well about 10^6^ CFU/mL, and incubated at 25 °C for 60 h to obtain the mature *S. flexneri* biofilm. After the mature biofilm was formed, the wells were gently washed with sterile PBS. The Na_2_SO_4_ solution was added as a background solution. After adjusting the desired initial pH, quantitative amounts of EGCG, FeSO_4_, and PDS were added in order. The reaction was terminated by adding excess Na_2_S_2_O_3_ at setting time intervals, and the reaction wells were washed with sterile water. The experiments were replicated at least 3 times in each group.

After the AOP treatment, the plate was stained using 0.1% crystal violet according to previous research [7]. After air drying, 1.5 mL of 95% ethanol was added to dissolve crystal violet, then the solution was transferred to a 96-well plate, and the absorbance value was measured at 570 nm (OD_570_). The biomass reduction rate and the relative percentage of biofilm formation were calculated by Equation (1) and Equation (2), respectively.

(1) The biomass reduction rate=100%−relative percentage of biofilm formation

(2) $$\mathrm{Relative}\;\mathrm{percentage}\;\mathrm{of}\;\mathrm{biofilm}\;\mathrm{formation}=\;\frac{{OD}_{570}(control\;group)-{OD}_{570}(experimental\;group)}{{OD}_{570}(control\;group)}\times 100\%$$

Further application was conducted with tap water instead of ultrapure water in order to assess the adaptability of the EGCG-enhanced system, and the other steps were the same as above.

A detailed description of the experimental process can be found in Text S2.

### 2.3. Determination of Effective Ferrous

The concentration of Fe^2+^ in the system was determined by 1,10-phenanthroline UV spectrophotometry [8]. Details are described in Text S3. The effective Fe^2+^ proportion was expressed as the ratio of C/C_0_, where C represents the actual Fe^2+^ concentration, and C_0_ denotes the initial introduced Fe^2+^ concentration. The experiments were replicated at least 3 times in each group.

### 2.4. Detection of Reactive Species

Ascorbic acid (AA) and tertiary butyl alcohol (TBA) were selected as quenchers for SO_4_^•−^ and ^•^OH, respectively [9,19]. Meanwhile, 1,4-Benzoquinone (p-BQ) and L-histidine were chosen as quenchers for ^•^O_2_^−^ and ^1^O_2_, respectively [20]. The experiments were replicated at least 3 times in each group.

Radicals generated in the system were further identified by electron paramagnetic resonance (EPR). 5,5-Dimethyl-1-pyrroline-N-oxide (DMPO) and 2,2,6,6-Tetramethylpiperidine (TEMP) were used as free radical trapping agents [20,21].

Methyl phenyl sulfoxide (PMSO) could be oxidized by Fe(IV)/Fe(V) to methyl phenyl sulfone (PMSO_2_), serving as a typical probe for detecting aqueous Fe(IV/V) species [22]. PMSO was then employed to detect aqueous Fe(IV/V) species [23].

A detailed description of the experimental process can be found in Text S4.

### 2.5. Microscopic Analysis

A field emission scanning electron microscope (FESEM) was applied for morphological observation. A confocal laser scanning microscope (CLSM) was used for detection of membrane integrity and cell status. Z-stack scanning was also performed using the CLSM, and 3D reconstruction and biofilm-related analysis were subsequently carried out with Imaris x64 9.0.1 software [24,25]. Details are described in Texts S5 and S6.

### 2.6. Composition Variation of Cell Membrane

The activated *S. flexneri* was processed using the Bacterial Membrane Protein Extraction Kit (Bestbia, Shanghai, China) according to the instructions. The composition of the cell membrane was subsequently analyzed using Fourier Transform Infrared (FTIR) Spectroscopy (INVENIO S, Bruker, Ettlingen, Germany). Data analysis was conducted using Origin 2024 software according to previous research [26]. Details are described in Text S7.

### 2.7. Determination of Typical Oxidative Stress Indicators

As important oxidative stress response indicators, total ROS and MDA were investigated. Total ROS levels were determined using the Reactive Oxygen Species Assay Kit (Beyotime, Shanghai, China) and MDA levels were measured using the Lipid Peroxidation MDA Assay Kit (Beyotime, Shanghai, China). Details are described in Text S8. The experiments were replicated at least 3 times in each group.

### 2.8. Biotoxicity Assay

After the AOP treatment, excess quencher was added to terminate the reaction. The reaction mixture was aspirated and filtered through a 0.45 μm membrane for acute toxicity testing using *V. fischeri*, with 20 g/L NaCl solution serving as the blank control [27]. Details are described in Text S9. The experiments were replicated at least 3 times in each group.

The inhibition rate was calculated using Equation (3):

(3) $$E\%=\frac{I_{0}-I}{I_{0}}\times 100\%$$

where *E* (%) represents the luminescence inhibition rate, and *I* and *I*_0_ denote the average luminescence values of test samples and blank controls after 15 min exposure, respectively.

### 2.9. Data Analysis

Data were analyzed with SPSS 23.0 using multifactor analysis of variance (ANOVA), and the F-test was employed. Statistical significance was set at *p* < 0.05, and high significance at *p* < 0.01. Results are expressed as mean ± SD, and figures were prepared with Origin 2024.

## 3. Results and Discussion

### 3.1. Biomass Variation Under Different Treatments

**Figure 2:**
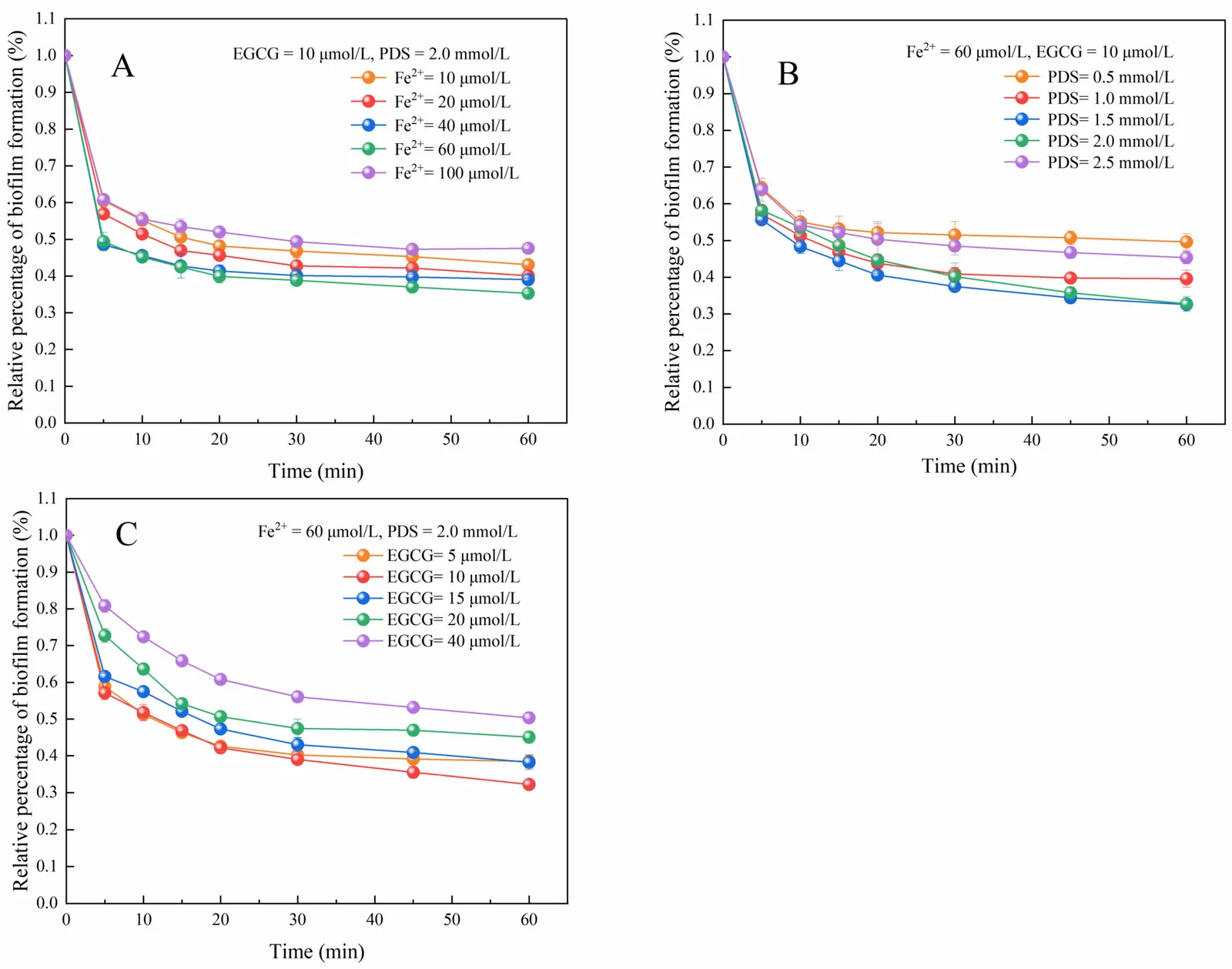
Biomass variation under different treatments in the EGCG/Fe^2+^/PDS system (pH = 3). (**A**) The effect of Fe, (**B**) the effect of PDS, (**C**) the effect of EGCG.

The biomass reduction rate after Fe^2+^/PDS system treatment achieved an average of 68.89 ± 0.52% at the dosage of 0.8 mmol/L Fe^2+^, 2.0 mmol/L of PDS and pH 3 (details are described in Text S10 and Figure S1). After the introduction of EGCG, the effects of Fe^2+^, PDS, and EGCG on the biomass variation are illustrated respectively in Figure 2. For the EGCG-enhanced system, similarly to the Fe^2+^/PDS system, a rapid reaction phase was presented for the first 10 min with a quick decrease in the relative percentage of biofilm formation, which reached equilibrium at about 30 min. Meanwhile, considering the cost-effectiveness, with the addition of EGCG, when the biomass reduction rate reached the peak of 67.76 ± 0.62%, the dosage of Fe^2+^, PDS and ECCG was 60 μmol/L, 1.5 mmol/L and 10 μmol/L respectively, indicating a 92.5% and 25% decrease in Fe^2+^ and PDS loading respectively, but with a very similar biomass reduction rate.

In the EGCG-enhanced system (Figure 2A), the biomass reduction rate first increased with the increase in the Fe^2+^ dosage, and the maximum reduction rate occurred at the Fe^2+^ concentration of 60 μmol/L. Then the biomass reduction rate showed a decreasing tendency with the increase in Fe^2+^. Undeniably, the increase in Fe^2+^ dosage would promote the activation of PDS, resulting in greater generation of SO_4_^•−^ (Equation (4)), while the excessive Fe^2+^ would trigger a side reaction (Equation (5)), which depletes the SO_4_^•−^ generated in the system [28] and affects the biomass reduction. A previous study suggested that when the Fe^2+^ concentration was too high, Fe^2+^ could react with SO_4_^•−^ and inhibit the degradation of aniline [29], and another survey also found that diuron degradation was maximized at neutral pH with an optimal Fe^2+^-to-PDS ratio, but decreased with excess Fe^2+^ [30]. This might suggest that in the EGCG-enhanced system, the appropriate concentration range of effective Fe^2+^ would help to achieve both highly efficient biomass reduction and fewer chemical inputs.

Fe^2+^ + S_2_O_8_^2−^ → SO_4_^•−^ + Fe^3+^ + SO_4_^2−^ (4)

Fe^2+^ + SO_4_^•−^ → Fe^3+^ + SO_4_^2−^ (5)

In the EGCG-enhanced system, the effect of increasing PDS dosage on biomass reduction also showed a similar variation to that of the Fe^2+^/PDS system. The highest biomass reduction rate was reached when the dosage of PDS was 1.5 mmol/L. The reason might be that several side reactions occurred when the dosage of PDS was excessive. It is common sense that more activated PDS might result in the production of more SO_4_^•−^. However, when the concentration of SO_4_^•−^ is too high, a self-quenching effect occurs, resulting in a meaningless consumption of those reactive species and a decrease in the concentration of SO_4_^•−^ (Equation (6)). Secondly, excess PDS (presented as S_2_O_8_^2−^) would also consume SO_4_^•−^ (Equation (7)), thus affecting the degradation [31]. Previous studies also indicated that excessive PDS in Fe^2+^/PDS inhibited the degradation of bisphenol A [32] and aniline [33]. It could be assumed that the excess PDS would compete for free radicals with the target pollutants, thus reducing the degradation rate.

SO_4_^•−^ + SO_4_^•−^ → S_2_O_8_^2−^ (6)

SO_4_^•−^ + S_2_O_8_^2−^ → S_2_O_8_^•−^ + SO_4_^2−^ (7)

The effect of EGCG on the biomass reduction rate is shown in Figure 2C. There was an increase in the biomass reduction rate when the dosage of EGCG was elevated from 5 μmol/L to 10 μmol/L. However, when the dosage of EGCG was more than 10 μmol/L, the biomass reduction rate of biofilm decreased. In previous investigations, similar tendencies of orange G [17] and atrazine [34] were also observed with the variation in EGCG concentration in an EGCG/Fe^2+^/PDS system. EGCG contains eight hydroxyl hydrogens, which would play an antioxidant role by providing H^+^. The neighboring phenolic hydroxyl group on EGCG could form complexes by ligand interaction with metal ions, thus promoting Fe^2+^/Fe^3+^ redox cycling [35,36].

Nevertheless, when the concentration of EGCG is too high, the biomass reduction efficiency of the system decreases rather than increases. This is due to the fact that EGCG is also a well-known free radical scavenger that sometimes reduces oxidative stress and ROS generation [34] through the Hydrogen Atom Transfer mechanism [37]. Specifically, EGCG could eliminate free radicals (R^•^) to produce stabilized compounds (RH) and phenoxyl radicals [38].

Notably, it should be addressed that the “optimal” conditions identified in this study were derived from selected concentrations of Fe^2+^, PDS, and EGCG, and do not accurately represent the “true optimum” for these AOPs where the synergistic interactions often occur. Further multivariate optimization and a comprehensive response surface analysis might be required if necessary.

### 3.2. The Effect of Initial pH

**Figure 3:**
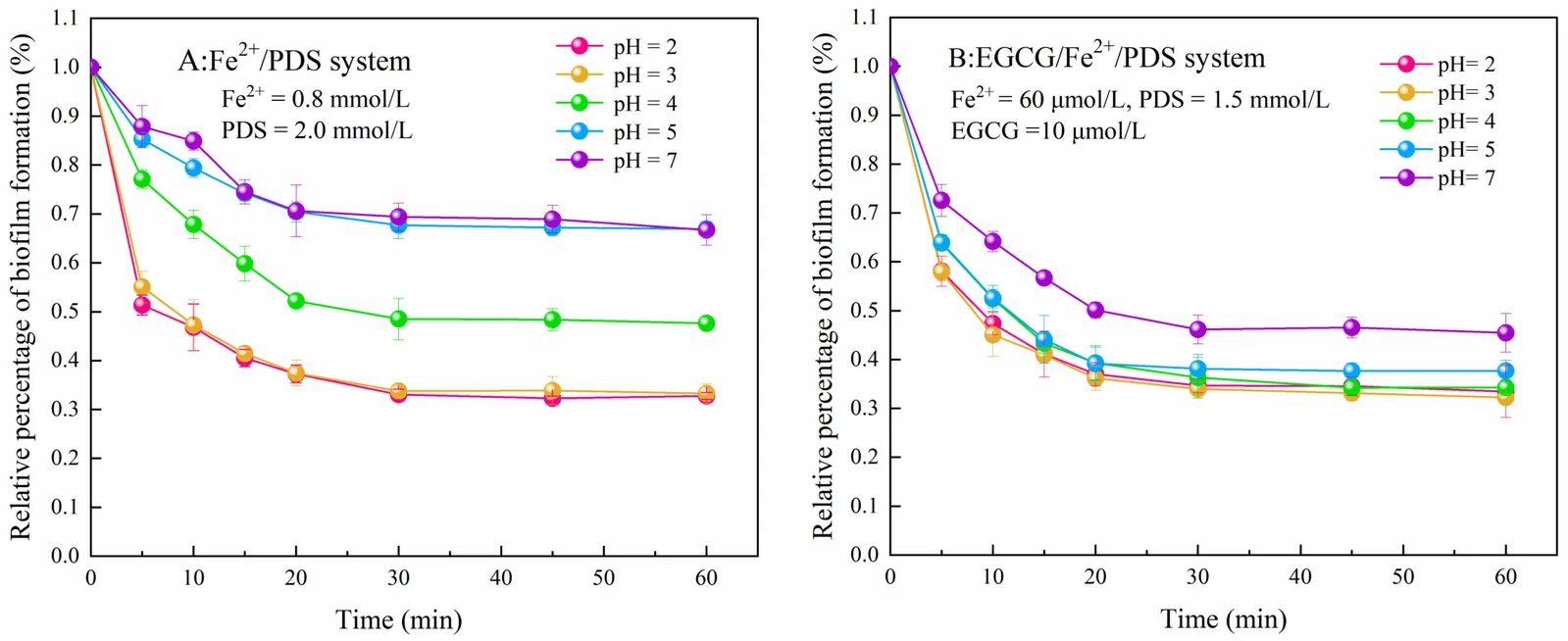
The effect of initial pH on biomass reduction. (**A**) Fe^2+^/PDS system, (**B**) EGCG/Fe^2+^/PDS system.

The effect of initial pH on biomass variation is shown in Figure 3. In the Fe^2+^/PDS system, the biomass reduction rate decreased markedly (from 67.22 ± 0.70% at pH 2 to 33.28 ± 3.11% at pH 7) as the pH increased. This decrement was attributed to the influence of pH on both the valence and phase state of iron, which consequently diminished the oxidative capacity of the system. This is consistent with previous findings, which showed decreased removal rates of iodoform [39], *Escherichia coli* and *Enterococcus faecalis* suspension [40] with the increase in initial pH.

Unlike the Fe^2+^/PDS system, the biomass variation in the EGCG-enhanced system showed a slight decreasing trend with the increase in initial pH (Figure 3B). It could be seen that at pH 5, the biomass reduction rate increased from 33.07 ± 1.54% in the Fe^2+^/PDS system to 62.32 ± 2.15% in the EGCG-enhanced system, reflecting an increase of 29.25 percentage points. Similarly, at pH 7, the biomass reduction rate increased from 33.28 ± 3.11% (without EGCG) to 54.53 ± 3.97% (with EGCG), indicating an enhancement of 21.25 percentage points. This observation suggested that the addition of EGCG contributed to the improved pH adaptability especially when pH was in the weakly acidic and neutral range. Even if the pH of the system was increased from 2 to 7, the biomass reduction rate in the enhanced system was only reduced by 11.99 percentage points.

### 3.3. Variation in Effective Ferrous Content

**Figure 4:**
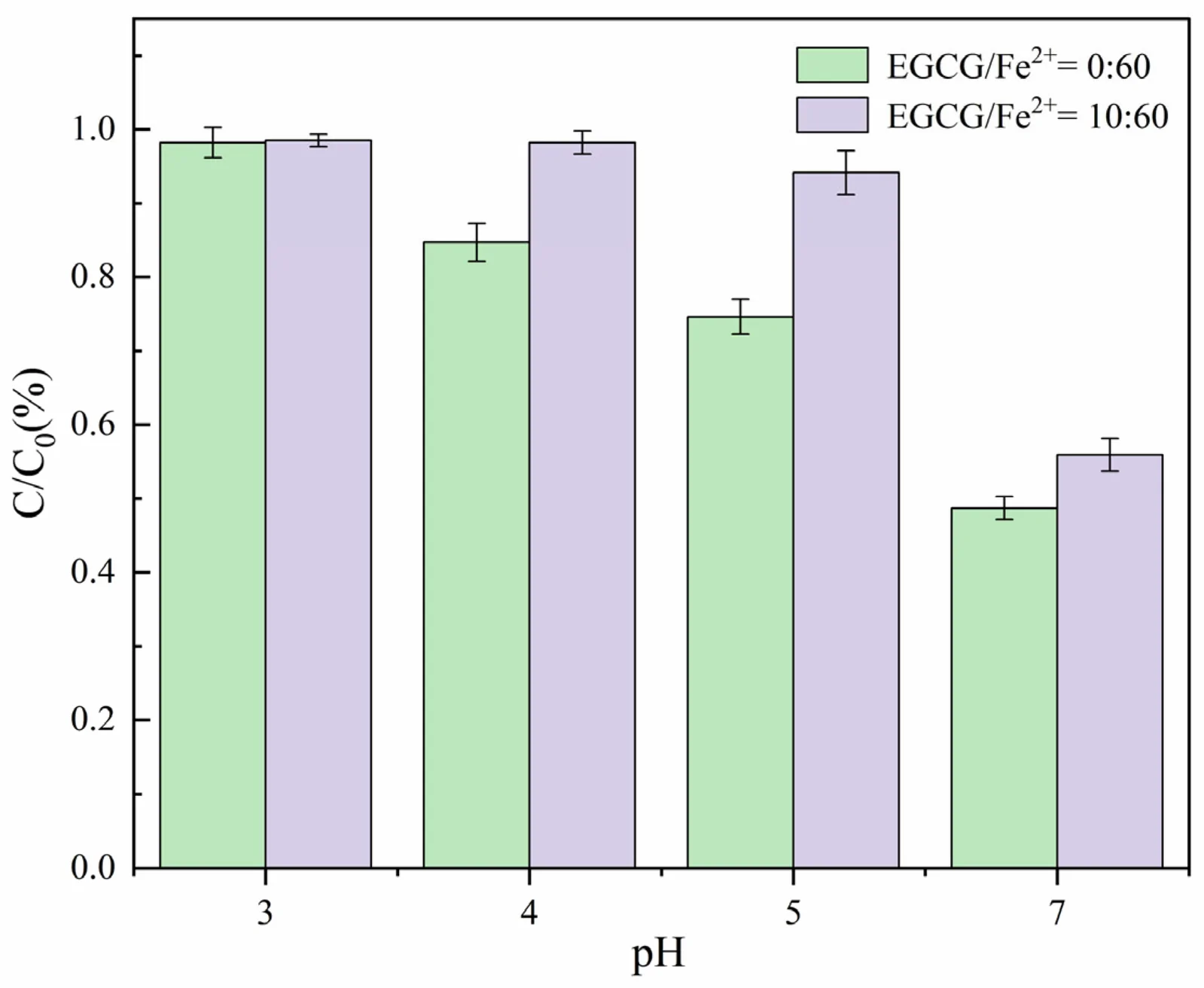
Effective Fe^2+^ concentration ratio in solution at different pH for the two systems.

The results of Fe^2+^ concentration ratio at different pH for both systems are shown in Figure 4. It is obvious that at pH 3, the values of C/C_0_ in both systems were nearly the same. As pH increased to 4 and 5, the effective Fe^2+^ concentration in the Fe^2+^/PDS system markedly decreased, while the decline in the EGCG-enhanced system was slight. Under the neutral-pH conditions, both systems exhibited an obvious decrease in the effective Fe^2+^ concentration, but the effective Fe^2+^ concentration in the enhanced system remained higher than that in the Fe^2+^/PDS system.

Generally speaking, in the Fe^2+^/PDS system, Fe^2+^ is easily oxidized to Fe^3+^ at high pH and precipitation might form. Once Fe^2+^ is oxidized to Fe^3+^, it might be hard to reduce to Fe^2+^. In previous studies, EGCG, functioning as an antioxidant and complexing agent, was indicated to lower the reduction energy barrier and improve the stability of Fe^2+^ [35]. Specifically, it was suggested that EGCG could react with both Fe^2+^ and Fe^3+^ to produce semiquinone complexes [34]; meanwhile, it could also reduce Fe^3+^ to Fe^2+^ via chelation, which further converts the generated semiquinone into quinone, and thereby promotes the Fe^2+^/Fe^3+^ redox cycle [16,36].

Combined with Figure 3 and Figure 4, a similar effect might be expected in the present study. It might be hypothesized that at higher pH (such as pH=7), more Fe might still maintain an effective dissolved status after the introduction of EGCG, resulting in a higher biomass reduction comparing with the Fe^2+^/PDS system.

### 3.4. Reactive Species Assessment

#### 3.4.1. Contribution of Common Reactive Species

**Figure 5:**
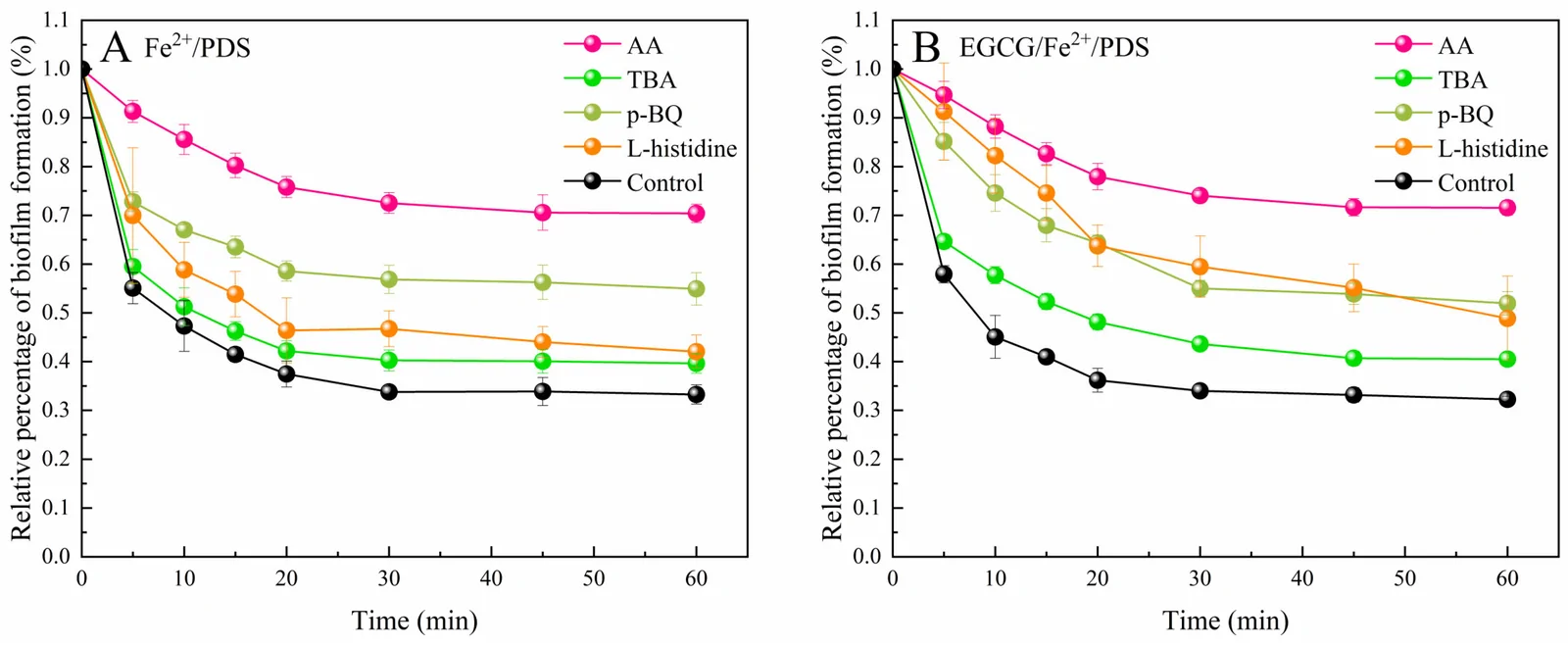
The influence of different quenching agents on biomass reduction. (**A**) Fe^2+^/PDS system: Fe^2+^ = 0.8 mmol/L, PDS = 2.0 mmol/L, pH = 3. (**B**) EGCG/Fe^2+^/PDS-enhanced system: EGCG = 10 μmol/L, Fe^2+^ = 60 μmol/L, PDS = 1.5 mmol/L, pH = 3.

In order to investigate whether the contribution to biomass reduction varies among different reactive species in the EGCG-enhanced system, quenching experiments were conducted and the results are shown in Figure 5 and Table S3.

It might indicate that ^•^OH, SO_4_^•−^, ^•^O_2_^−^ and ^1^O_2_ existed in both systems but SO_4_^•−^ might be the dominant active species contributing to biomass reduction. In addition to this, the composition of reactive species in the system seemed to not be changed obviously by the introduction of EGCG except ^1^O_2_.

**Figure 6:**
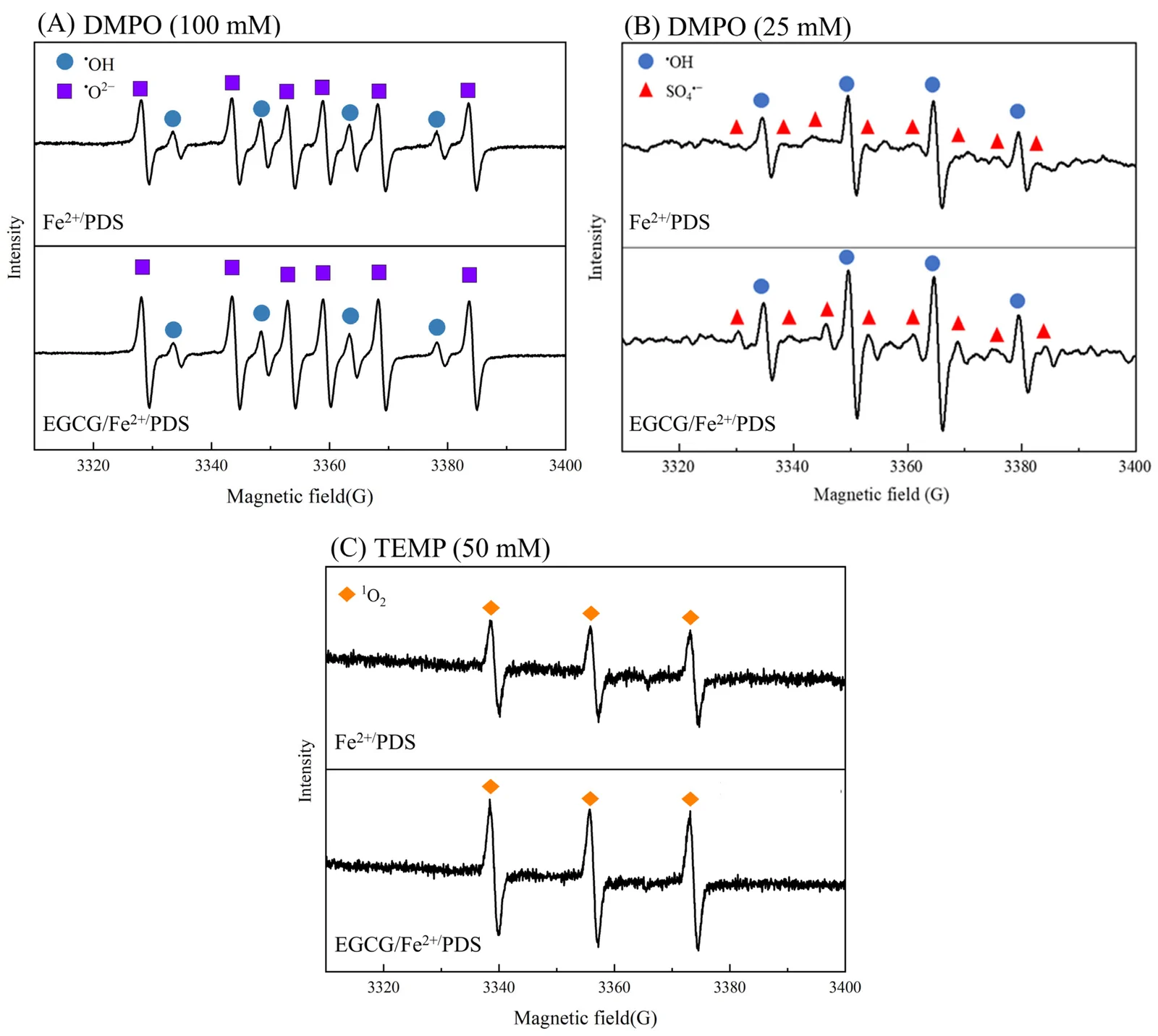
EPR spectra of different systems. (**A**) EPR signals of DMPO (100 mM), (**B**) EPR signals of DMPO (25 mM), (**C**) EPR signals of TEMP (50 mM).

However, the quenching experiments showed relatively low selectivity [41]. When alcohol species are selected as quenchers in the PDS-based AOPs, they might interrupt the reaction between ^•^OH, SO_4_^•−^, and PDS, thereby interfering with the results [42]. Thus, DMPO and TEMP were used as trapping agents to further characterize the active species generated in this study. Figure 6A presents the results obtained with 100 mM DMPO, revealing the detection of ^•^OH and ^•^O_2_^−^ in both systems. When 100 mM DMPO was employed as the spin-trapping agent, the signal peaks corresponding to ^•^OH and ^•^O_2_^−^ were observed to be relatively intense, which could result in the masking of the weaker signal peak of SO_4_^•−^ [43]. Therefore, after quenching ^1^O_2_, 25 mM DMPO was used for reidentification of SO_4_^•−^ signals, as shown in Figure 6B. Figure 6C indicates the presence of ^1^O_2_ in both systems. Based on the visualization comparison of EPR, it was suggested that the EGCG-enhanced system slightly enhanced the peak signals of ^•^OH, ^•^O_2_^−^, SO_4_^•−^ and ^1^O_2_. Similar EPR spectra were obtained when the EGCG/Fe^2+^/PDS-enhanced system was applied for atrazine degradation, with the signal intensity being more pronounced in the enhanced system compared to the Fe^2+^/PDS system [34]. It was also found that a mixed green tea extract could promote the generation of ^•^O_2_^−^, ^•^OH and SO_4_^•−^ during the oxidative degradation of trichloropropane in the Fe^2+^/PDS system, with ^•^OH playing the primary role in the degradation process [44].

#### 3.4.2. High-Valent Iron in EGCG-Enhanced System

**Figure 7:**
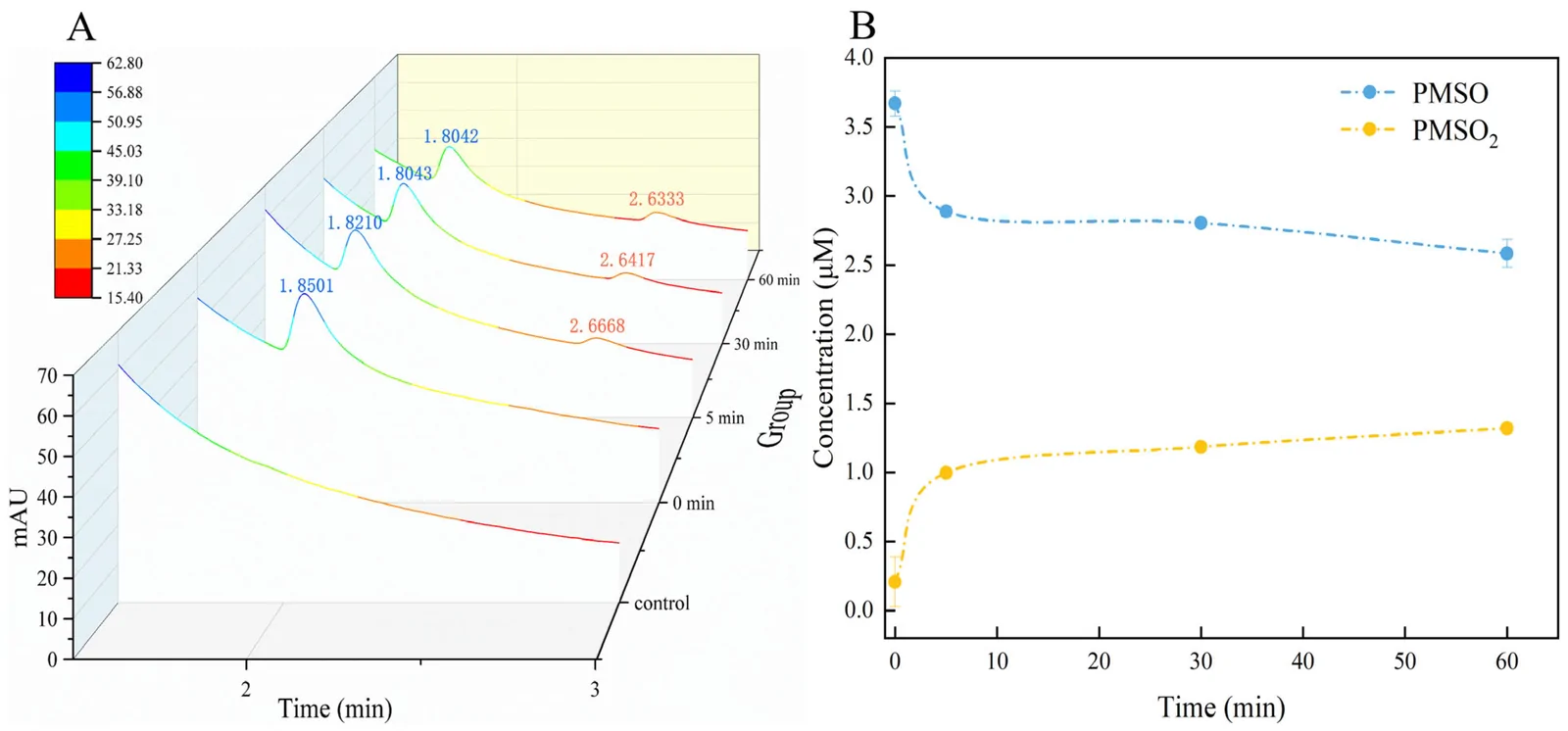
HPLC determination results of PMSO in EGCG/Fe^2+^/PDS systems. (**A**) Liquid chromatograms of PMSO and PMSO_2_, (**B**) concentration changes in PMSO and PMSO_2_ in EGCG/Fe^2+^/PDS systems.

High-valent iron might act as an effective oxidant in Fe^2+^/PDS systems [45]. Thus, whether the introduction of EGCG would affect the presence of high-valent iron was examined. PMSO was detected in the EGCG-enhanced system with a retention time of approximately 1.8 min, while PMSO_2_ was identified in the 5, 30, and 60 min treatment groups with a retention time of around 2.6 min (in Figure 7A). PMSO peak intensity decreased with the treatment time extension, whereas PMSO_2_ peak intensity progressively increased (in Figure 7). The results confirmed PMSO-to-PMSO_2_ conversion in the EGCG-enhanced system, suggesting that high-valent iron species formed in the EGCG-enhanced system. A previous investigation demonstrated Fe(IV)’s presence in Fe^2+^/PDS systems through PMSO_2_ formation [45], while another study verified Fe(IV) involvement in PMSO degradation within MBQ (Methyl-p-benzoquinone, MBQ)/Fe^3+^/PDS systems [46], both supporting the current study.

Li et al. found that in the Fe^2+^/PDS system, the production of Fe(IV) is mediated by SO_4_^•−^, rather than by PDS or ^•^OH. Fe(IV) is primarily formed through oxygen transfer from PDS to Fe^2+^, highlighting the critical role of SO_4_^•−^ in the generation of Fe(IV) [47]. This might also explain the sharp decrease in biomass reduction rate after the addition of AA which is the main quencher of SO_4_^•−^ (in Figure 5 and Figure 6).

It could be seen that in the present EGCG-enhanced system, ^•^OH, SO_4_^•−^, ^•^O_2_^−^, ^1^O_2_ and high-valent iron species might work together to reduce biomass. However, high-valent iron could undergo side reactions with Fe^2+^, PDS, H_2_O_2_, H_2_O, etc., which might complicate the entire system. Further studies are still needed to analyze the specific proportion relating to each active species and their contribution to biomass reduction.

### 3.5. Biofilm Morphology and Cell Status Observation

**Figure 8:**
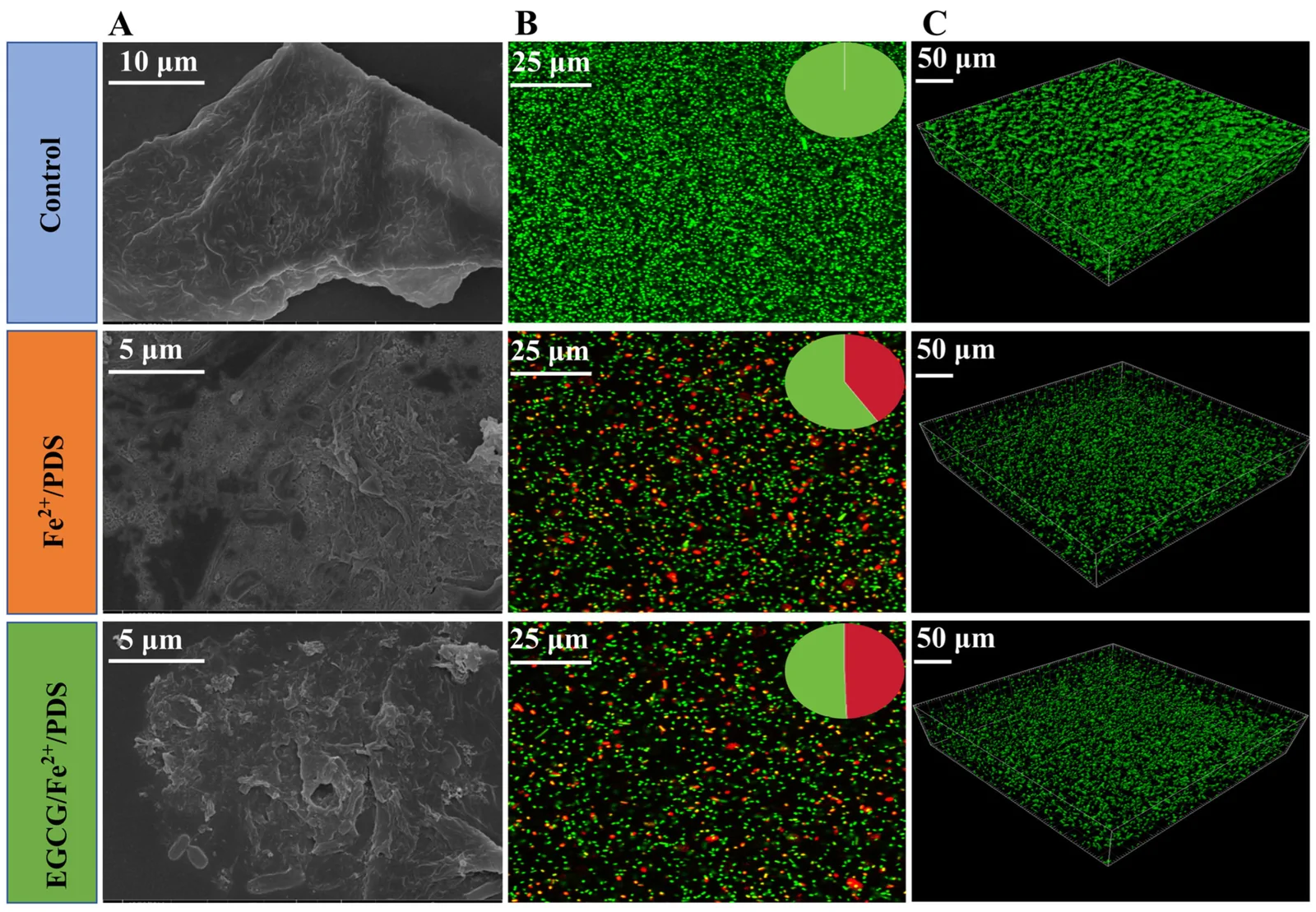
FESEM and CLSM images before inactivation, and after Fe^2+^/PDS and EGCG-enhanced treatment. (**A**) FESEM images, (**B**) CLSM images (red represents dead bacteria; green represents relative bacteria), (**C**) 3D reconstruction images.

The morphological changes in biofilms are presented in Figure 8A. Due to copious amounts of EPS, the bacteria formed a cohesive biofilm, exhibiting good integrity, a dense structure, and a certain thickness. After treatment by the Fe^2+^/PDS system, the structure of the biofilm was disrupted to some extent, and the thickness of the biofilm was reduced. When treated by the EGCG-enhanced system, the biofilm exhibited more pronounced wrinkling, and bacteria rupture can be observed in the figure. This indicated that the EGCG-enhanced system had obvious destructive effects on the biofilm structure.

In order to further assess the viability of bacterial cells inside the biofilm after the treatment of the EGCG-enhanced system, the biofilm was stained using SYTO 9 and PI at different reaction times. The CLSM images corresponding to merged fluorescence channels and 3D reconstruction images corresponding to SYTO 9 fluorescence channels are shown in Figure 8B and Figure 8C respectively. The results of the optical density value of CLSM images and the data of 3D reconstruction are shown in Table 1.

Combined with Figure 8B and Table 1, it could be seen that there were no red fluorescent dots in the picture at 0 min, and the bacteria within the biofilm were in a good state of survival. With a prolonged reaction time, the density of bacteria in the biofilm decreased and the number of dead bacteria increased, indicating that the EGCG-enhanced system could inactivate the bacteria in the biofilm. At the end of the reaction (60 min), the bacterial density within the biofilm was reduced by 49.38% for the EGCG-enhanced system. Meanwhile, for the Fe^2+^/PDS system, the bacterial density was reduced by 40.67%. This might indicate that the EGCG-enhanced system could promote the decrease in both the total live cell density and the overall bacterial density in the biofilm. As shown in Figure 8C, after treatment with both systems, both the area and volume of the 3D reconstruction biofilms markedly reduced.

### 3.6. FTIR Analysis of Bacterial Membrane Proteins

**Figure 9:**
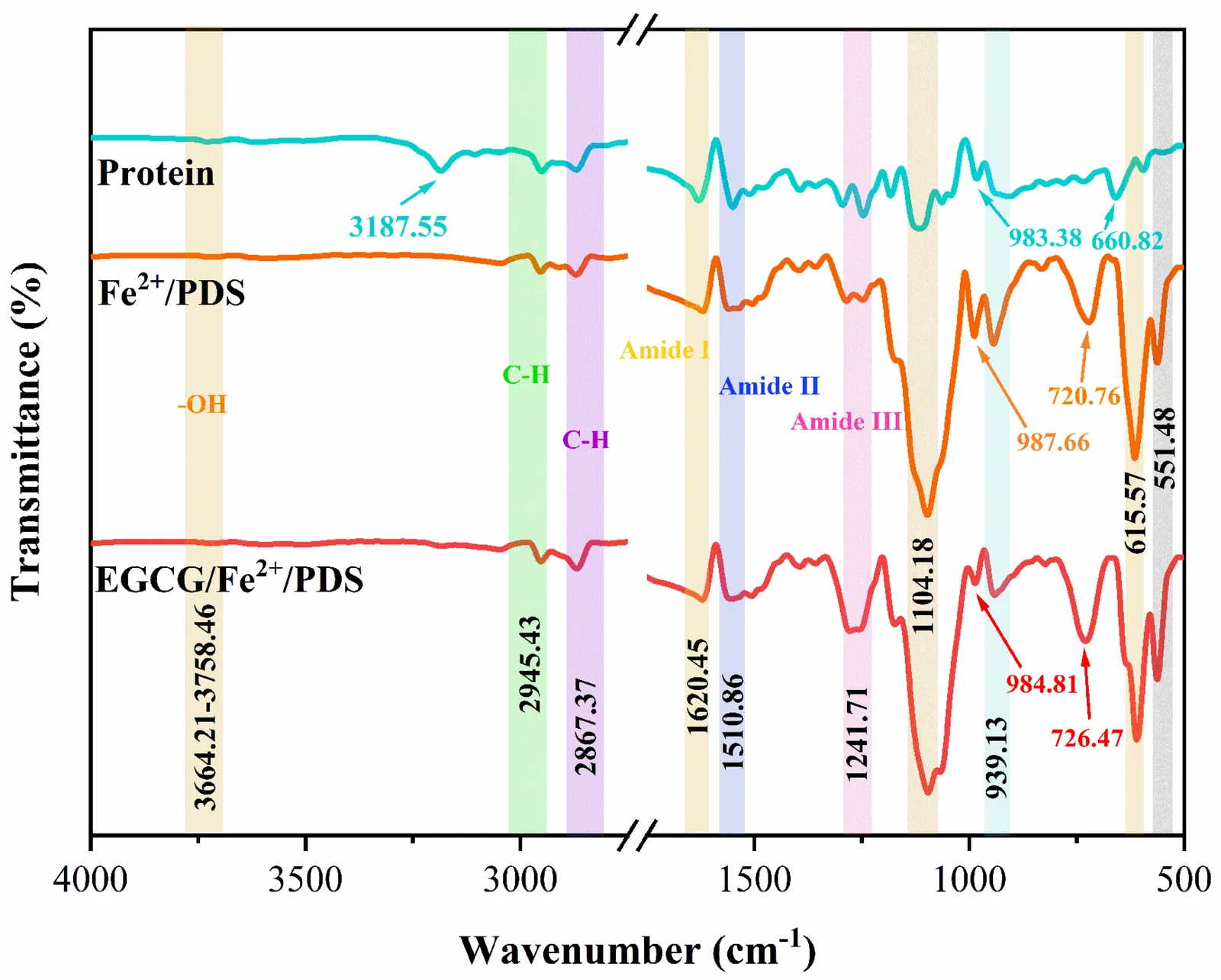
Infrared spectra of membrane protein monomers and complexes after treatment of membrane proteins with both systems.

The variation in bacterial membrane proteins is shown in Figure 9.

A broad absorption feature was observed near 3188 cm^−1^, which may contain overlapping contributions from hydrogen-bonded phenolic hydroxyl (-OH) and amino (-NH) stretching vibrations [48]. Compared to the protein group (control group), the FTIR spectrum of both the Fe^2+^/PDS system and EGCG-enhanced system showed the near disappearance of the characteristic peaks for (-OH) and (-NH) groups. This phenomenon was primarily attributed to the coordination reaction between Fe^2+^ and phenolic hydroxyl (-OH) groups. Moreover, high-valent iron was also shown to preferentially target compounds with greater electron-donating capacity [49]. Obvious variations in the peak intensities at 1241.71 cm^−1^ (amide III) for the EGCG-enhanced system were observed, which were tentatively assigned to C-N bonds of peptide linkages. This might be due to the structural modifications caused by EGCG–protein complexation, which was consistent with previous reports demonstrating EGCG’s capacity to alter protein conformation [50]. Moreover, both systems exhibited a sharp increase at 1104.18 cm^−1^. This is likely due to the oxidation capacity and histidine, C-H, and C-N vibrations, along with possible additional overlaps from amino acid side chains and other functional groups [51].

Both the Fe^2+^/PDS system and the EGCG-enhanced system exhibited enhanced intensities of characteristic peaks at 939.31, 615.57, 551.48 cm^−1^, and approximately 980.00 cm^−1^ compared to the protein group. This phenomenon might result from increased bond strength and consequent elevation of vibrational frequencies. Moreover, compared to the Fe^2+^/PDS system, the EGCG-enhanced system exhibited similar peak distribution (from 500 to 750 cm^−1^) but different peak positions and intensities, suggesting similar changes in protein structure may occur in both systems. In this experiment, membrane proteins were extracted independently and subsequently introduced into two reaction systems to simulate the transformation process of proteins in the biofilm. This approach just constitutes an artificial proxy for what happens in a living biofilm and could not exactly reflect the real situation.

### 3.7. Biofilm Oxidative Stress Response

**Figure 10:**
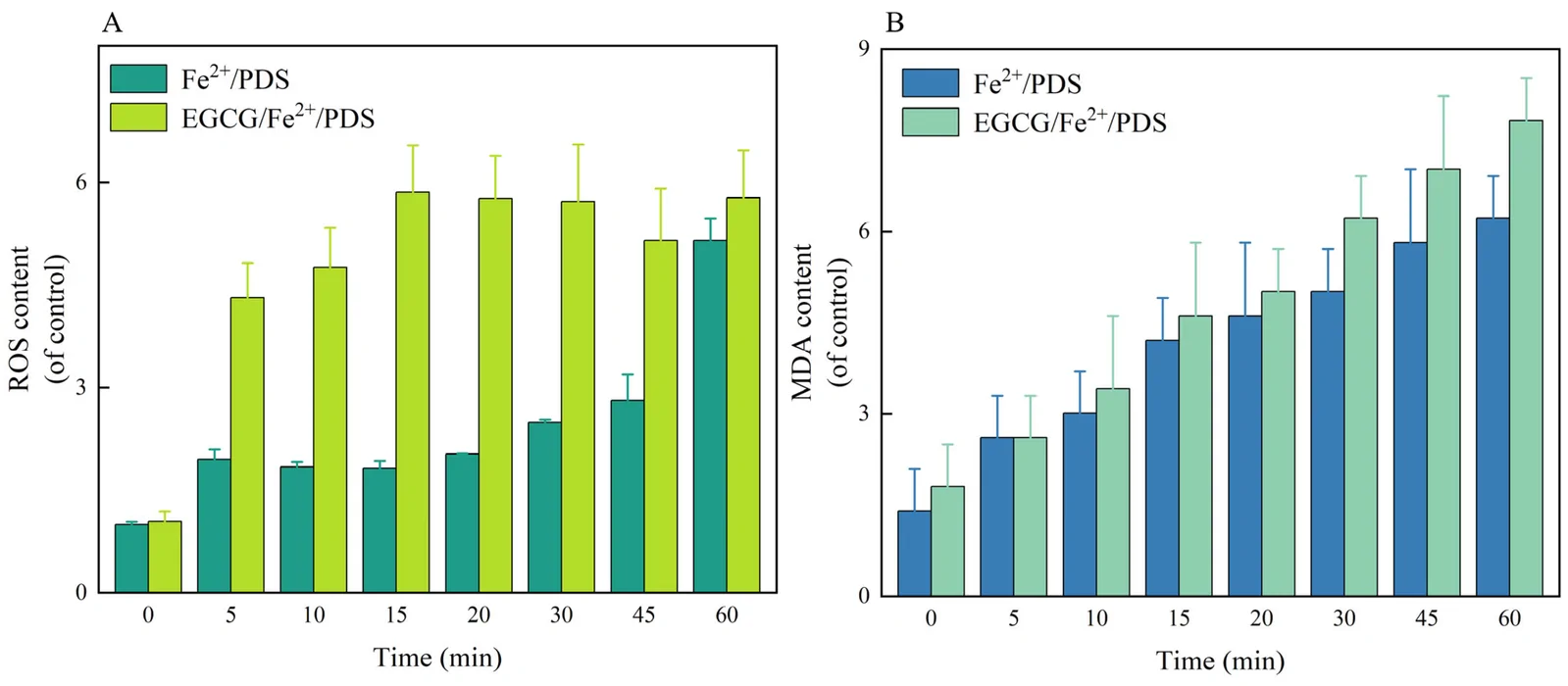
ROS and MDA levels in Fe^2+^/PDS and EGCG/Fe^2+^/PDS systems: (**A**) ROS; (**B**) MDA.

As shown in Figure 10A, the total ROS levels of biofilms in both systems generally rose over time. For the Fe^2+^/PDS system, the total ROS level reached its peak which was 5.15 times the initial level. Compared to the Fe^2+^/PDS system, the EGCG-enhanced system suggested a rapid increase followed by a plateau phase, and the level increased to 5.77 times its initial value after 60 min. These results indicated that the EGCG-enhanced system rapidly generated substantial ROS to enhance biomass reduction. This was consistent with the results mentioned in Figure 2 and Figure 3, which showed a faster increase in biomass reduction efficiency during the first 20 min for the EGCG-enhanced system.

Figure 10B shows MDA levels in biofilms for both the Fe^2+^/PDS and EGCG/Fe^2+^/PDS systems, which exhibited an overall upward trend over time and reached the peak at 60 min. It could be seen that the addition of EGCG promoted MDA production.

These findings collectively indicated that the EGCG-enhanced system generated more ROS and MDA than the Fe^2+^/PDS system, thereby enhancing oxidative stress in *S. flexneri* biofilms to cause structural damage and achieve more efficient biomass reduction.

### 3.8. Function Hypothesis of EGCG

Combined with the results of Section 3.3 focusing on effective Fe^2+^ and Section 3.5 focusing on the cell status, the observation leaves open the possibility that external environmental stimuli acted to raise membrane permeability, perhaps accelerating subsequent intracellular iron uptake [52]. This process might lead to the intracellular accumulation of ROS-dependent lipid peroxides and induce ferroptosis-like bacterial death [53]. Recent evidence has shown that phenolic compounds can chelate iron and markedly promote ^•^OH generation via the Fenton reaction, resulting in lipid peroxidation and ferroptosis-like death in *E. coli* [54]. Such ferroptosis-like death has been recognized as a novel programmed cell death in microbes, characterized by iron overload, ROS accumulation and membrane lipid peroxidation [55]. Moreover, EGCG has been suggested as a ferroptosis sensitizer [56]. In the present study, a similar process was hypothesized based on the associated observation of increased ROS generation and lipid peroxidation, but direct ferroptosis-specific evidence such as intracellular iron accumulation and changes in key biomolecular markers is still needed.

**Figure 11:**
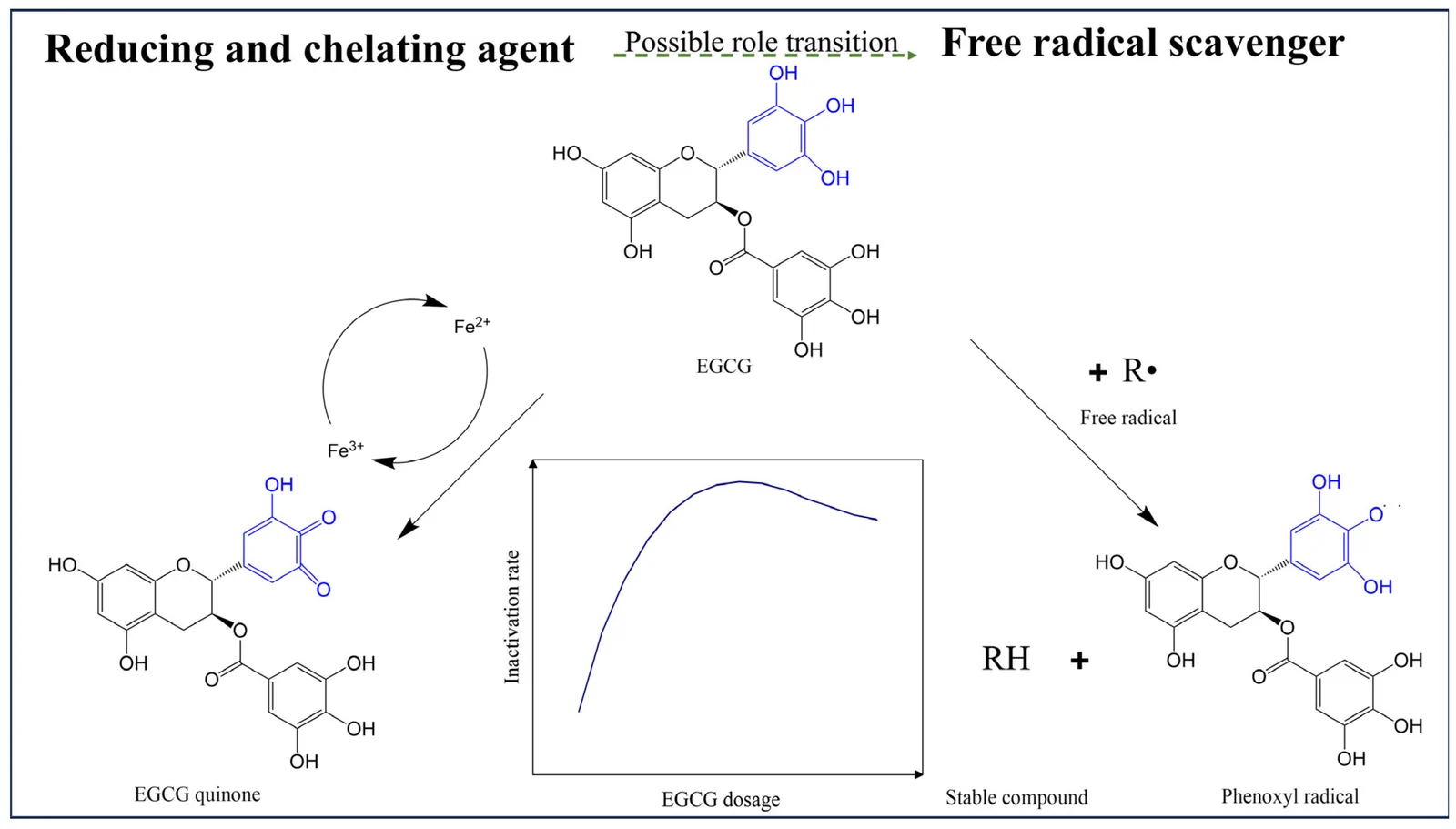
Possible impact of EGCG concentration on the enhanced system.

It is hypothesized that the role of EGCG is multifaceted in the present study: its antibacterial property, reducibility, and complexation ability may all contribute to biomass reduction and bacterial inactivation. First, EGCG could inhibit the growth of microorganisms directly [7]. Secondly, EGCG could form complexes by ligand interaction with metal ions, facilitating Fe^2+^/Fe^3+^ redox cycling and accelerating PDS activation. The activated PDS could trigger the generation of free radicals and non-free radicals, promoting biomass reduction and cell inactivation. Thirdly, EGCG might act as a ferroptosis sensitizer and it might induce ferroptosis indirectly. However, when the concentration of EGCG is too high (as shown in Figure 2C), it could also act as a free radical scavenger. Then the impact of EGCG shifts with concentration, as shown in Figure 11. Further comprehensive studies are still needed.

### 3.9. Biotoxicity Assessment

**Figure 12:**
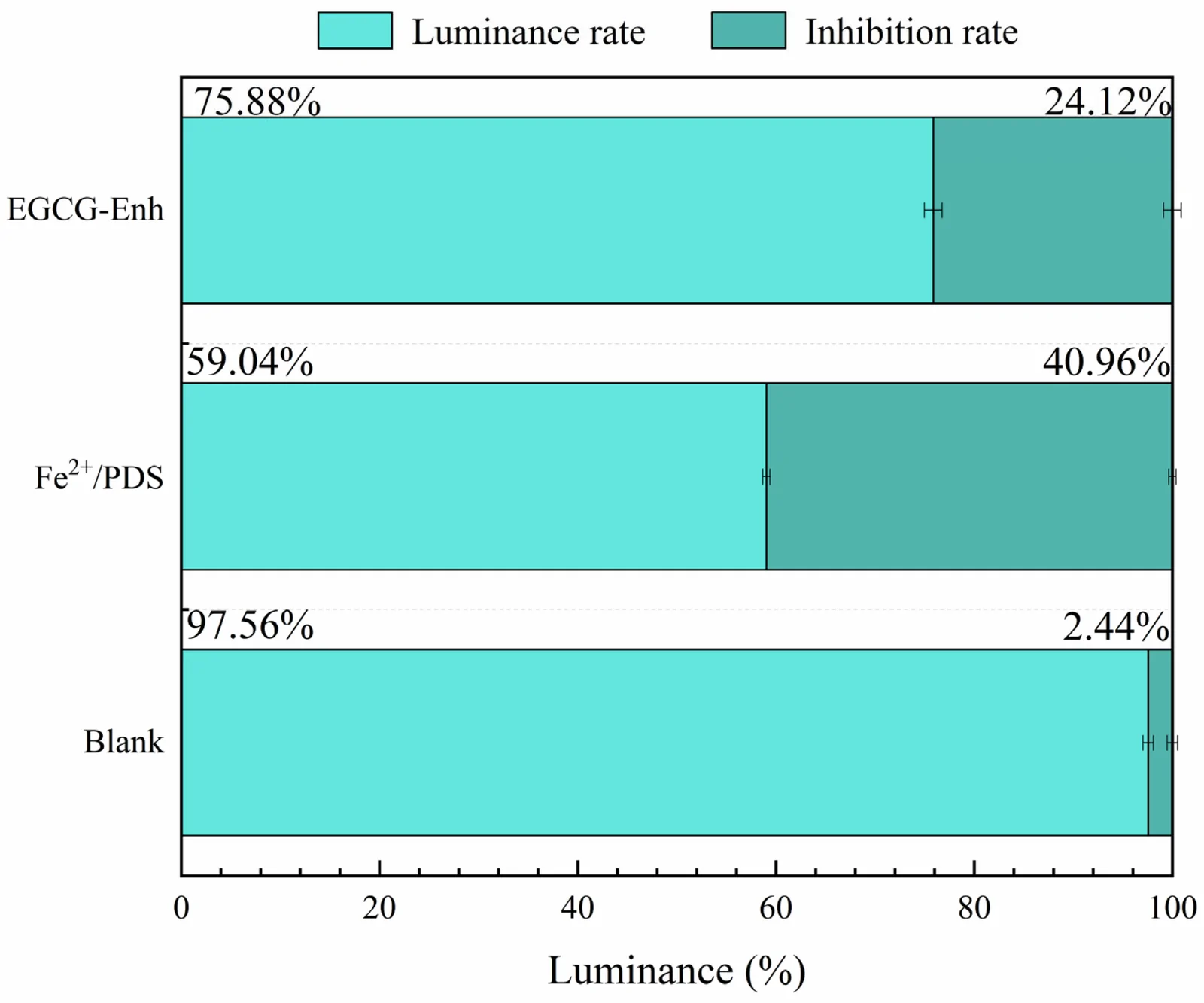
Acute biotoxicity of Fe^2+^/PDS and EGCG/Fe^2+^/PDS systems (Blank: PBS solution; Fe^2+^/PDS: Fe^2+^ = 0.8 mmol/L, PDS = 2.0 mmol/L, pH = 3; EGCG-Enh: EGCG/Fe^2+^/PDS: EGCG = 10 μmol/L, Fe^2+^ = 60 μmol/L, PDS = 1.5 mmol/L, pH = 3. Triplicate experiments with duplicate parallel samples per group).

*V. fischeri* was employed to assess acute biotoxicity, with luminescence inhibition rates indicating toxicity levels as shown in Figure 12. Blank control groups exhibited luminescence rates of 97.56%, whereas both the Fe^2+^/PDS and EGCG-enhanced systems increased toxicity post-treatment. The Fe^2+^/PDS system showed a 59.04% luminescence rate, corresponding to an average 38.52 percentage point reduction in luminescence relative to blanks. For the EGCG-enhanced system, a 75.88% luminescence rates reflected an average 21.68 percentage point luminescence reduction comparing to the blanks. This suggested the EGCG-enhanced system decreased the biotoxicity comparing with the Fe^2+^/PDS system. Microorganisms and their metabolic products have been suggested to be important precursors of disinfection byproducts (DBPs) including C-DBPs and N-DBPs [57]. In previous research, EGCG was suggested to potentially reduce toxicity through chelation mechanisms leveraging its inherent oxidative/reductive properties [16]. Moreover, the oxidation of Fe(IV)/Fe(V) might also reduce acute toxicity [58].

### 3.10. Tap Water Application

**Figure 13:**
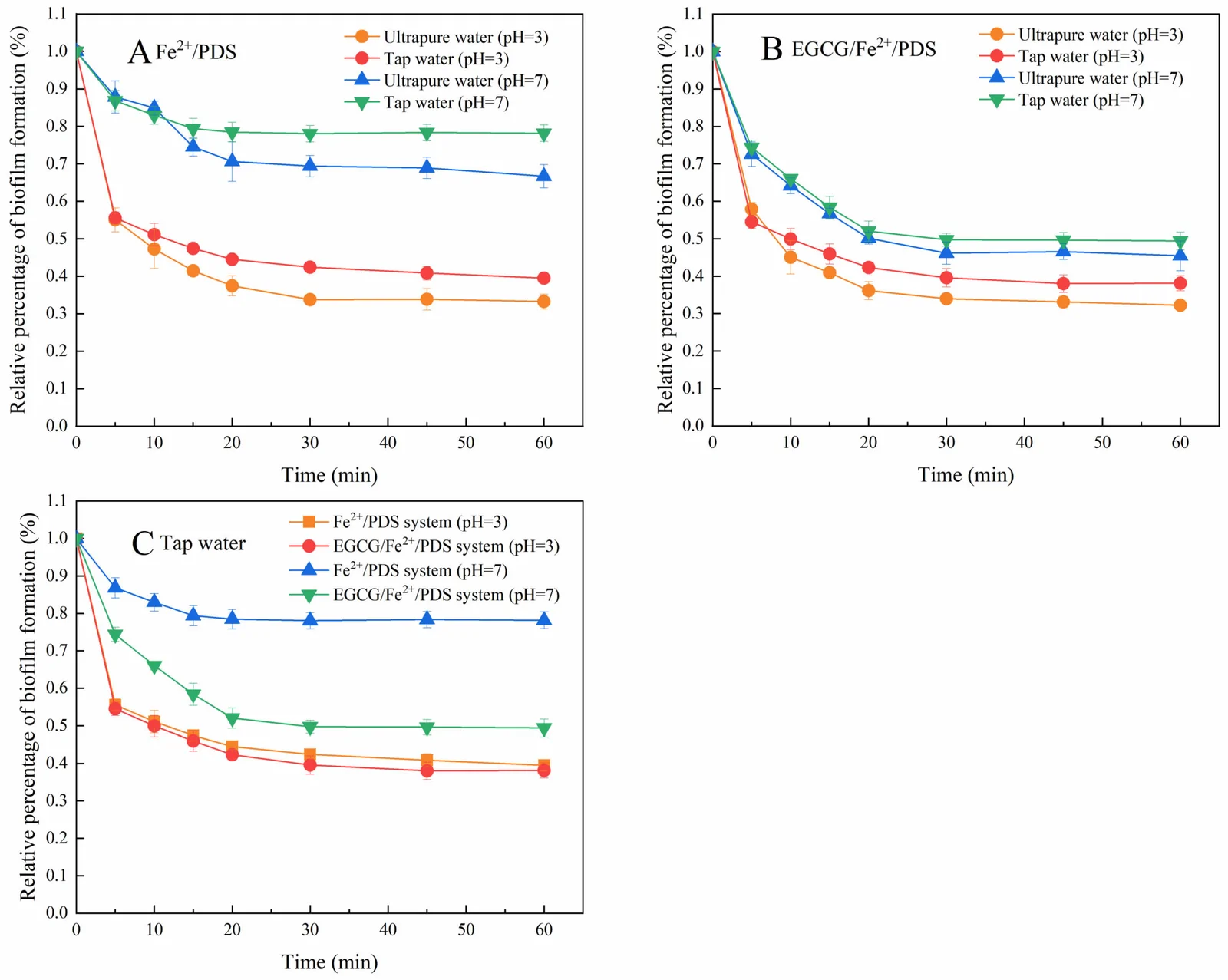
Comparison of Fe^2+^/PDS system and EGCG-enhanced system on biomass reduction in different water matrices: (**A**) Fe^2+^/PDS system, (**B**) EGCG/Fe^2+^/PDS-enhanced system, (**C**) tap water application with different pH.

Inorganic anions, natural organic matter (NOM), etc., in a real water matrix might have an adverse influence on AOPs. Therefore, the biomass reduction effects in ultrapure and tap water were compared at pH 3 and 7, respectively (as shown in Figure 13 and Table S4), and a similar decreasing tendency for the relative percentage of biofilm formation was suggested for the two water matrices with the pH variation.

In the Fe^2+^/PDS system (Figure 13A), for ultrapure water, after a 60 min reaction, the biomass reduction rate decreased by 35.61 percentage points when the pH was increased from 3 to 7. Meanwhile, in tap water, the biomass reduction rate decreased by 38.66 percentage points. These results indicated an interference of the water matrix and a limited scope for Fe^2+^/PDS system application.

In the EGCG-enhanced system (Figure 13B), similarly to the Fe^2+^/PDS system, the relative percentage of biofilm formation in both ultrapure and tap water decreased with the extension of reaction time at both pH 3 and pH 7. When pH increased from 3 to 7, after a 60 min reaction, the biomass reduction rate decreased by 13.23 percentage points in ultrapure water, while it declined by 11.29 percentage points in tap water, representing a slightly smaller reduction.

The application of the Fe^2+^/PDS and EGCG-enhanced systems in tap water at pH 3 and 7 is summarized more visually in Figure 13C. Interestingly, it could be seen that the biomass reduction rate of both systems in tap water is very close at pH 3. However, when pH reached 7, the biomass reduction rate in the EGCG-enhanced system was 28.73 percentage points higher than that in the Fe^2+^/PDS system. This indicates that the EGCG-enhanced system is less affected by other matrices in the tap water and possesses good adaptability in the present study. As described in Section 3.2, the Fe^2+^/PDS system is more susceptible to pH variation. Besides pH, another important reason for the much lower reduction rate of the Fe^2+^/PDS system might be the presence of inorganic anions and NOM in tap water. Previous studies have shown that inorganic anions (Cl^−^, NO_3_^−^, HCO_3_^−^, H_2_PO_4_^−^, etc.) could negatively affect Fe^2+^/PDS [6,34]. On one hand, the inorganic anions would react with both Fe^2+^ and Fe^3+^ to form precipitates, resulting in a decrease in effective Fe^2+^. On the other hand, the inorganic anions might also scavenge SO_4_^•−^ and ^•^OH directly [59,60,61]. In addition, NOM is also known as a strong radical scavenger. The electronic groups presented in the NOM structure are easily reduced by SO_4_^•−^ and ^•^OH [62,63].

The EGCG-enhanced system is less susceptible to interference in tap water than the Fe^2+^/PDS system in this study. It might be partially attributed to the complexation of EGCG, which would stabilize effective Fe^2+^ to activate PDS, thereby compensating for free radical consumption induced by interfering substances to a certain extent. It is worth noting that the pH and water matrix complexity (such as NOM, inorganic ions, and so on) may still differ in various practical water matrices [64,65,66]. Therefore, further studies focusing on different water matrices would be required.

## 4. Conclusions

EGCG, as a natural extract, is beneficial to human health. However, EGCG is not well utilized and this causes a huge waste of resources. After the introduction of EGCG to the Fe^2+^/PDS system in this study, it was suggested that the dosage of Fe^2+^ and PDS might be largely reduced with comparable biomass reduction and biofilm inactivation resulting from the increased utilization efficiency of Fe^2+^ and decreased interference of the tap water matrix. The stronger oxidation stress generated after EGCG introduction might also be helpful for biomass reduction and bacteria inactivation. This study provides new ideas for the control of biofilm contamination and new perspectives for green disinfection. Moreover, such utilization of tea products in this study might be a promising application for agricultural resources.

## Figures and Tables

**Table 1:** Optical density value of CLSM images and data of 3D reconstruction images.

Reaction Condition	Control	Fe^2+^/PDS System	EGCG-Enhanced System
Treatment time (min)	0	60	60
Total optical density values	0.1446	0.0858	0.0732
Relative bacterial density	100%	59.33%	50.62%
Area (µm^2^)	1411	111	117
Volume (µm^3^)	2162	78.7	84

(1) Total optical density values (ODn) under different reaction conditions. (2) Relative bacterial density = ODn/OD0 × 100%, where OD0 is the total optical density value in 0 min, which is the bacterial density in the biofilm before treatment. (3) Area and volume data obtained from 3D reconstruction using Imaris x64 9.0.1.

## Data Availability

The original contributions presented in this study are included in the article/Supplementary Material. Further inquiries can be directed to the corresponding author.

## Supplementary Materials

The following supporting information can be downloaded at: https://www.mdpi.com/article/10.3390/microorganisms14081717/s1, Text S1. Materials. Text S2. *S. flexneri* biofilm cultivation and AOPs treatment. Text S3. Determination of effective ferrous. Text S4. Detection of reactive species. Text S5. Morphological observation. Text S6. Detection of cell status. Text S7. Composition variation assessment of cell membrane. Text S8. Determination of Total ROS and MDA. Text S9. Biotoxicity assessment. Text S10. Biomass reduction efficiency under different factors in traditional Fe^2+^/PDS system. Table S1. Multifactor ANOVA on the effect of reaction conditions on biomass reduction in Fe^2+^/PDS system. Table S2. Multifactor ANOVA on the effect of reaction conditions on biomass reduction in EGCG/Fe^2+^/PDS system. Table S3. Biomass reduction rate of Fe^2+^/PDS and EGCG-enhanced systems with different quenching agents. Table S4. Biomass reduction rate of Fe^2+^/PDS and EGCG-enhanced systems in different water matrix. Figure S1. Biomass variation under different treatment in the Fe^2+^/PDS system. Reference [67] is cited in the supplementary materials.
