# Supplementary material for: EGCG-Enhanced Ferrous-Activated Peroxydisulfate Processes for Shigella flexneri Biofilm Inactivation: Efficacy, Underlying Mechanisms, and Practical Implications
Source: Microorganisms. 2026 Aug 5;14(8):1717. doi: 10.3390/microorganisms14081717 (PMC13515669; doi:10.3390/microorganisms14081717)
Supplement: Supplementary file 1 [file microorganisms-14-01717-s001.zip › microorganisms-4406782-supplementary.pdf]

Supplementary Material for：

EGCG enhanced ferrous-activated peroxydisulfate processes for *Shigella flexneri* biofilm inactivation: efficacy, underlying mechanisms, and practical implications

Xuan Liu [1,2], Yu Chen [1,2], Zheng Ji [1,2,*], Yeyue Zhang [1,2], Yini Zhang [3], Yuru Wang [1,2] and Minrui Li [1,2]

1 School of Geography and Tourism, Shaanxi Normal University, Xi'an 710119, China; liuxuan2023@snnu.edu.cn (X.L.); 20242419@snnu.edu.cn (Y.C.); zhangyeyue0223@163.com (Y.Z.); wangyuru@snnu.edu.cn (Y.W.); liminrui503@126.com (M.L.)

2 International Joint Research Centre of Shaanxi Province for Pollutants Exposure and Eco-Environmental Health, Xi'an 710119, China

3 School of Environment, Northeast Normal University, Changchun 130117, China; zoeyyy97@163.com

* Correspondence: jizheng@snnu.edu.cn; Tel.: +86-13572260810

List of captions:

Text S1. Materials 3

Text S2. *S. flexneri* biofilm cultivation and AOPs treatment 4

Text S3. Determination of effective ferrous 6

Text S4. Detection of reactive species 7

Text S5. Morphological observation 9

Text S6. Detection of cell status 10

Text S7. Composition variation assessment of cell membrane 11

Text S8. Determination of Total ROS and MDA 12

Text S9. Biotoxicity assessment 13

Text S10. Biomass reduction efficiency under different factors in traditional Fe^{2+}/PDS system 14

Table S1. Multifactor ANOVA on the effect of reaction conditions on biomass reduction in Fe^{2+}/PDS system 15

Table S2. Multifactor ANOVA on the effect of reaction conditions on biomass reduction in EGCG/Fe^{2+}/PDS system. 16

Table S3. Biomass reduction rate of Fe^{2+}/PDS and EGCG-enhanced systems with different quenching agents 17

Table S4. Biomass reduction rate of Fe^{2+}/PDS and EGCG-enhanced systems in different water matrix 18

Figure S1. Biomass variation under different treatment in the Fe^{2+}/PDS system 19

References 20

Text S1. Materials

Shigella flexneri (*S. flexneri*, ATCC 12022) was purchased from Hope Bio-Technology Co., Ltd. *Vibrio fischeri* (*V. fischeri*, ATCC 25918) was purchased from China Center of Industrial Culture Collection (CMCC). LB was purchased from Beijing Aobox Bio-Technology Co., Ltd. PBS buffer was purchased from Servicebio Technology (Wuhan) Co. Ltd. EGCG (purity ≥ 98%) was purchased from Shanghai Yuanye Bio-Technology Co., Ltd. $Na_2S_2O_8$ (analytically pure), formic acid (HPLC grade) and acetonitrile (HPLC grade) were purchased from Tianjin Damao Chemical Reagent Factory. $FeSO_4 \cdot 7H_2O$ (analytically pure) was purchased from Fuchen (Tianjin) Chemical Reagent Co., Ltd. Na_2SO_4 (analytically pure), $Na_2S_2O_3$ (analytically pure), and 95% ethanol (analytically pure) were purchased from Tianjin Tianli Chemical Reagents Ltd. LIVE/DEAD™ BacLight™ Bacterial Viability Kits (Cat. No. L7012) was purchased from Thermo Fisher scientific Co., Ltd. The Bacterial Membrane Protein Extraction Kit (Cat. No. BB-3151-50T) was purchased from Shanghai BestBio Biotechnology Co., Ltd. The Reactive Oxygen Species Assay Kit (Cat No. S0033S) and the Lipid Peroxidation MDA Assay Kit (Cat No. S0131S) were purchased from Beyotime Biotechnology Co., Ltd. Crystal violet (analytically pure) was purchased from Tianjin Komeo Chemical Reagents Co., Ltd. DMPO (analytically pure) was purchased from Tohito Chemical Technology (Shanghai) Co., Ltd. PMSO (analytically pure) was purchased from Macklin Biochemical Technology Co., Ltd.

Text S2. *S. flexneri* biofilm cultivation and AOPs treatment

A ceramic bead with *S. flexneri* was taken out from the cryopreservation tube and added into LB broth, then incubated at 37 °C 150 rpm for 18 h. The bacterial culture medium was then dipped into SS agar medium according to the dilution-plate method and then incubated at 37 °C for 24 h. The single colony was picked up and inoculated into LB broth at 37 °C 150 rpm for 12 h to complete the strain activation [1].

The activated bacterial suspension was diluted with 1/2 LB broth to make the initial concentration of the bacterial solution in each reaction well to about 10^6 CFU/mL, and incubated at 25 °C for 60 h to obtain the mature *S. flexneri* biofilm [2].

After the mature biofilm was formed, the remained LB broth was gently discarded. Then 1.5 mL of sterile PBS buffer was added to wash the residual suspended bacteria away and repeated 3 times. The Na_2SO_4 solution at a concentration of 5 mmol/L was added to the 12-well plate as a background solution, and after adjusting the desired initial pH with prepared H_2SO_4 and NaOH solution, quantitative amounts of EGCG, $FeSO_4$, and PDS were added in order. The two primary AOPs systems employed in this mechanistic study (Fe^{2+}/PDS system and the EGCG/Fe^{2+}/PDS system) were optimized through preliminary experiments. The optimal reagent dosages were determined as follows: for the Fe^{2+}/PDS system, [Fe^{2+}] = 0.8 mmol/L, [PDS] = 2.0 mmol/L, and pH = 3, meanwhile, for the EGCG/Fe^{2+}/PDS system, [EGCG] = 10 μmol/L, [Fe^{2+}] = 60 μmol/L, [PDS] = 1.5 mmol/L, and pH = 3. Conditions for other systems were indicated in the corresponding figure legends. The reaction was terminated by adding excess sodium thiosulfate to the wells at setting

time intervals, the reaction solution was discarded and the reaction well was washed with 1.5 mL of sterile water and repeated three times. The inoculated wells without any reagents of the advanced oxidative reaction system were used as a positive control, the uninoculated wells with the addition of 1/2 LB broth alone were used as a negative control, and the uninoculated wells with the same conditions that the advanced oxidative reaction system was applied as the background control, and the experiments were replicated at least 3 times in each group.

After the AOPs treatment, the 12-well plate was dried at 60 °C for 15 min to fix the biofilm. Each well was stained with 1.5 mL of 0.1% crystal violet solution for 30 min. After staining, the plates were gently washed three times with sterile water to remove excess dye and air-dried at RT. After drying, 1.5 mL of 95% ethanol was added to each well to dissolve the crystal violet. The solution was transferred to a 96-well plate, and the absorbance value was measured at 570 nm (OD_{570}) using the microplate reader (Spectra Max ABS Plus, Shanghai Molecular Devices Co., Ltd).

The real water matrix application experiment was carried out further with tap water instead of ultrapure water for the AOPs treatment in order to assess the adaptability of the EGCG-enhanced system, and the other steps were the same as above.

Text S3. Determination of effective ferrous

The concentration of Fe^{2+} in the system was determined by 1,10-phenanthroline UV spectrophotometry [3]. With 5mM Na_2SO_4 solution used as the background, the initial pH was adjusted to 3, 4, 5, or 7, followed by the sequential addition of 0 or 10 μM EGCG and 60 μM $FeSO_4$ to initiate the reaction. After of the reaction, the Fe^{2+} concentration was determined. 2 mL of the solution to be measured was added into a 5 mL vial, then 2 mL of ortho phenanthroline solution (1.5 g/L, pH 2.5) was added, mixed thoroughly. The absorbance value of the mixed solution was measured at 510 nm by UV spectrophotometry (TU-1901, Beijing Purysis General Instrument Co., Ltd). The effective Fe^{2+} proportion was expressed as the ratio of C/C_0, where C represents the actual Fe^{2+} concentration, and C_0 denotes the initial added Fe^{2+} concentration.

Text S4. Detection of reactive species

Text S4.1 Quenching experiment

Ascorbic acid (AA) and tertiary butyl alcohol (TBA) were selected as free radical quenching agents. According to the previous research, the reaction rate of TBA with $^{\bullet}OH$ is relatively fast, while its reactivity towards $SO_4^{\bullet-}$ is low (k[TBA, $SO_4^{\bullet-}$]=9.1×10^5 $M^{-1}\cdot s^{-1}$, k[TBA, $^{\bullet}OH$]=$(3.8\sim7.6)\times10^8$ $M^{-1}\cdot s^{-1}$). Therefore, TBA is primarily employed for quenching $^{\bullet}OH$ and AA is utilized for quenching both $SO_4^{\bullet-}$ and $^{\bullet}OH$ [4,5]. The Fe^{2+}/PDS system both with and without EGCG received additions of 2.0 mmol/L AA or 4.0 mmol/L TBA, respectively, while the control group did not have free radical quencher introduced. 2.0 mmol/L 1,4-Benzoquinone (p-BQ) was chosen as the quencher for $^{\bullet}O_2^-$ and 2.0 mmol/L L-histidine for $^1O_2$ [6] respectively. The biofilm was then quantified by crystal violet staining at 5, 10, 15, 20, 30, 45, and 60 min, respectively. The absorbance value was measured at 570 nm by UV spectrophotometer. The biomass reduction rate of biofilm and the relative percentage of biofilm formation were calculated by Eq. 1 and Eq. 2, respectively.

Text S4.2 Electron paramagnetic resonance test

Radicals generated in the system were identified by electron paramagnetic resonance (EPR). 5,5-Dimethyl-1-pyrroline-N-oxide (DMPO, 25 mM and 100 mM respectively) and 2,2,6,6-Tetramethylpiperidine (TEMP, 50 mM) were used as free radical trapping agents [6-8]. DMPO and TEMP were added as spin traps to the

Na_2SO_4 background solution with the preset pH, and then the mixture was separately formulated into the Fe^{2+}/PDS system (Fe^{2+} = 0.8 mmol/L, PDS = 2.0 mmol/L, pH = 3) and the EGCG-enhanced system (EGCG = 10 μmol/L, Fe^{2+} = 60 mol/L, PDS = 1.5 mmol/L, pH = 3). After 2 min, 1.5 mL samples were drawn into capillaries, wiped clean, and placed in quartz tubes. The sample were tested in the EPR spectrometer (Bruker E500, Bruker Germany). EPR settings: center field 3356.40 G, sweep width 100 G, modulation 100 kHz/2.0 G, sweep time 80 s, microwave 9.42 GHz/20 mW. The free radicals generated in the system were characterized according to the spectrum obtained. The EPR spectra were baseline-corrected to eliminate background drift and noise, resulting in smooth original signal traces. Nevertheless, the absence of an internal standard precluded quantification of radical species.

Text S4.3 Detection of high-valent Fe species

PMSO was added to the reaction well in advance, followed by the treatment of EGCG-enhanced system. Excess quencher was then introduced to terminate the reaction, and the reaction mixture was aspirated, filtered through a 0.22 μm membrane, and sonicated for 30 min for degassing. PMSO and $PMSO_2$ were analyzed using UHPLC (Vanquish^{TM} UHPLC, Thermo Fisher Scientific, USA) with a mobile phase consisting of 0.1% formic acid in water and 0.1% formic acid in acetonitrile mixed in an 80:20 ratio, ultrapure water as the control group. Both compounds were detected at a UV wavelength of 230 nm [9].

Text S5. Morphological observation

The sterilized silicon wafers (10 mm×10 mm) were first placed into the 12-well plate. Then subsequent inoculation operations and the AOPs treatment (Fe^{2+}/PDS system and EGCG-enhanced system) of mature biofilm were carried out as described in section 2.2. Then, the silicon wafers were cleaned by ultrapure water and placed in 10 mL centrifuge tubes. Glutaraldehyde (2.5%) was added to make the wafers completely submerged, and the wafers were cultured at 4 °C overnight. After that, the wafers were washed with PBS buffer, followed by sequential dehydration with the gradient of ethanol-water solution with ratios of 50, 70, 80, 90, and 100% respectively. After dehydration, the biofilm samples were dried using the vacuum freeze dryer (LGJ-10, Sihuan Furui Keyi Technology Development (Beijing) Co., Ltd). The dried biofilm samples were sprayed with gold, then the morphology was observed using the field emission scanning electron microscope (FESEM, Nava NanoSEM 450, FEI Company, USA).

Text S6. Detection of cell status

A laser confocal petri dish was applied here for further observation. The biofilm was cultured and treated as described in section 2.2. Biofilm samples were stained using LIVE/DEAD^{TM} BacLight^{TM} Bacterial Viability Kits after washing with sterile water. The SYTO 9 and PI mixing solution was prepared according to the instructions and 500 μL of the mixing solution was added to the laser confocal dish. After staining for 15 min at RT in dark, the plates were washed three times with 0.9% saline to remove the excess dye solution. After washing, the anti-quench sealer was dripped in and photographed using the Confocal laser scanning microscope (CLSM) (TCSSP8, Leica, Germany). The CLSM micrographs were constructed by combining the images acquired from the SYTO 9 and PI staining channels. Z-stack scanning was performed using CLSM, and fluorescence images were acquired at 5 μm intervals over a Z position range of 3900-4000 μm. Three-dimensional reconstruction and biofilm-related analysis (area and volume) were subsequently carried out with Imaris x64 9.0.1 software [10,11]. The fluorescence intensity of the bacterial cells in the images was then statistically analyzed using ImageJ software. The Optical density (OD) values of the images obtained at each reaction stage were calculated by ImageJ to quantify the cell densities. The total optical density value represented the density of bacteria in biofilms after treatment with different inactivation conditions, and the percentage of red fluorescence represented the inactivation rate of bacteria in biofilms.

Text S7. Composition variation assessment of cell membrane

The activated *S. flexneri* was processed using the Bacterial Membrane Protein Extraction Kit according to the instruction. The obtained membrane protein samples were subjected to reactions under optimal conditions with Fe^{2+}/PDS system and EGCG-enhanced systems, respectively, for 60 min. Samples without any advanced oxidation systems were served as the blank control. After treatment, all samples were freeze-dried for 48 h to obtain the powdered samples, which were subsequently analyzed using Fourier Transform Infrared (FTIR) Spectroscopy (INVENIO S, Bruker, Germany) with an ATR sampling accessory. All data was collected using OPUS software. Data acquisition was performed at 4 cm^{-1} resolution, accumulating 64 scans over a spectral range of 4000-500 cm^{-1}. Each sample was analyzed in triplicate at least. Data analysis was conducted using Origin software according to previous research [12].

Text S8. Determination of Total ROS and MDA

Total ROS levels were determined using the Reactive Oxygen Species Assay Kit. 1 mL of 10 μmol/L DCFH-DA probe was added to the mature *S. flexneri* biofilms, followed by incubation at 37 °C for 30 min and subsequent AOPs treatment as described in Section 2.2. After treatment, the biofilms were resuspended in sterile PBS, and the fluorescence intensity was measured at 488 nm excitation and 525 nm emission wavelengths using a fluorophotometer (RF-6000, Shimadzu, Japan).

MDA levels were measured using the Lipid Peroxidation MDA Assay Kit. After the AOPs treatment, samples were resuspended in sterile PBS, transferred to centrifuge tubes, sonicated for 20 min, and centrifuged at 1000 × g for 10 min. The supernatant was mixed with pre-prepared MDA detection working solution, incubated in the boiling water bath for 15 min, then cooled to RT. After centrifugation at 1000 × g for 10 min, the supernatant was collected, and absorbance at 532 nm was measured using a microplate reader (KD-810A, Cody Share, China).

Text S9. Biotoxicity assessment

After the AOPs treatment, excess quencher was added at 60 min to terminate the reaction. The reaction mixture was aspirated and filtered through a 0.45 μm membrane for acute toxicity testing using *V. fischeri*, with 20 g/L NaCl solution serving as the blank control.

Cultivation of *V. fischeri*, preparation of culture media, and preservation methods strictly followed the standardized procedures outlined in ISO 11348-1:2007(E). A single colony was inoculated into 50 mL liquid medium and cultured at 20 °C with 180 rpm shaking for 20 h until OD_{575} reached 1.2, indicating the logarithmic growth phase. The bacterial suspension was diluted with NaCl solution (20 g/L), and luminescence intensity was measured using a single-tube multimode detector (Modulus™, Promega, USA), confirming stability between 3-4 million RLU for 2-3 min. Subsequently, 100 μL of *V. fischeri* suspension was added to each well. Immediately after inoculation, the 96-well plate was shaken for 30 s in a microplate luminometer (Centro LIA LB 962, Berthold Technologies, Germany) to ensure thorough sample contact, followed by 15 min air exposure before luminescence measurement [13].

The inhibition rate was calculated using Eq.3:

$$E(\%)=\frac{(I_0-I)}{I_0}\times 100\% \qquad \text{(Eq.3)}$$

Where E(%) represents the luminescence inhibition rate, and I and I_0 denote the average luminescence values of test samples and blank controls after 15 min exposure, respectively.

Text S10. Biomass reduction efficiency under different factors in traditional Fe^{2+}/PDS system

In the traditional Fe^{2+}/PDS system, although PDS is the main oxidant, the oxidative ability could be reduced significantly without the activator of Fe^{2+}. Therefore, the effect of Fe^{2+} and PDS dosage on the reduction of biomass in the Fe^{2+}/PDS system was investigated first. The results are shown in Figure S1 It could be seen that the relative percentage of biofilm formation decreased rapidly in the first 10 min, which is a fast-response phase, and then the equilibrium was reached around 30 min in both sets of experiments (as shown in Figure S1A and Figure S1B). According to Figure S1A, it could be seen that the biomass reduction efficiency of the Fe^{2+}/PDS system increased significantly when the Fe^{2+} dosage was gradually increased from 0.2 mmol/L to 0.8 mmol/L, and then decreased as the Fe^{2+} dosage continued to increase. That is, under the experimental condition of Figure S1A. As could be seen from Figure S1B, similar to the variation of Fe^{2+} dosage on biomass reduction efficiency, the increase of PDS dosage on biomass reduction first increased and then decreased. Under the experimental condition of Figure S1B. The reduction of biomass by the Fe^{2+}/PDS system achieved an average reduction rate of 68.89 ± 0.52% at the dosage of 0.8 mmol/L Fe^{2+}, 2.0 mmol/L of PDS and pH 3.

Table S1. Multifactor ANOVA on the effect of reaction conditions on biomass reduction in Fe^{2+}/PDS system

Dependent variable	Influencing factor	df	Mean square	F	*p*
Biomass reduction rate	Fe^{2+} dosage	4	0.005	19.196	<0.01
	PDS dosage	4	0.014	50.016	<0.01
	Initial pH of solution	4	0.122	497.191	<0.01

**p*<0.05, significant; *p*<0.01, highly significant.

Table S2. Multifactor ANOVA on the effect of reaction conditions on biomass reduction in EGCG/Fe^{2+}/PDS system.

Dependent variable	Influencing factor	df	Mean square	F	*p*
Biomass reduction rate	Fe^{2+} dosage	4	0.014	31.249	<0.01
	EGCG dosage	4	0.021	46.851	<0.01
	PDS dosage	4	0.024	53.549	<0.01
	Initial pH of solution	4	0.01	21.784	<0.01

*p<0.05, significant; p<0.01, highly significant.

Table S3. Biomass reduction rate of Fe^{2+}/PDS and EGCG-enhanced systems with different quenching agents

Quencher		TBA	AA	p-BQ	L-histidine
biomass reduction rate (t = 60 min)	Fe^{2+}/PDS	60.37 ± 2.01%	29.61 ± 1.87%	45.09 ± 3.33%	57.96 ± 3.41%
	EGCG-enhanced	59.49 ± 1.31%	28.46 ± 1.48%	48.06 ± 2.38%	51.14 ± 8.67%

Table S4. Biomass reduction rate of Fe^{2+}/PDS and EGCG-enhanced systems in different water matrix

t= 60 min		Fe^{2+}/PDS		EGCG-enhanced	
Water type		Ultrapure	Tap	Ultrapure	Tap
pH	3	68.89 ± 0.52%	60.50 ± 1.44%	67.76 ± 0.62%	61.86 ± 2.04%
	7	33.28 ± 3.11%	21.84 ± 2.22%	54.54 ± 3.97%	50.57 ± 2.39%

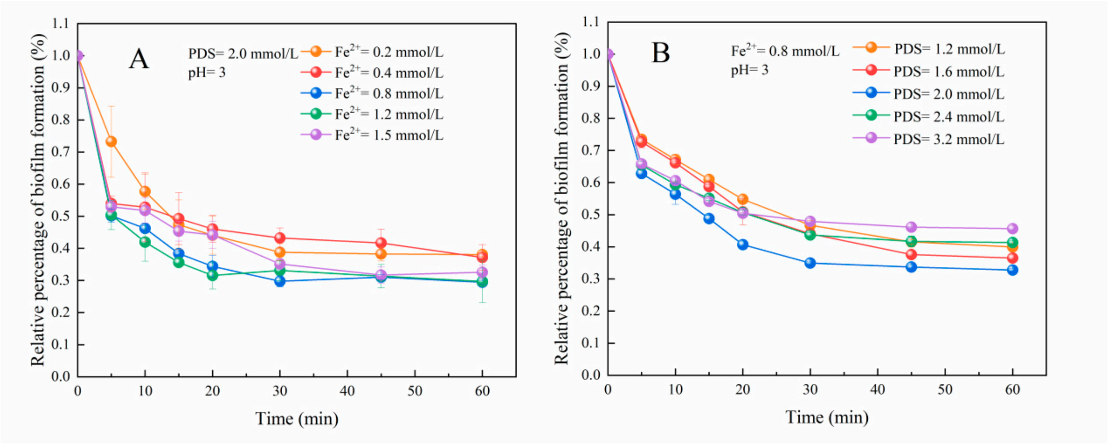

Figure S1. Biomass variation under different treatment in the Fe^{2+}/PDS system (pH = 3): (A) The effect of Fe^{2+} dosage. (B) The effect of PDS dosage.

References：

1.Zhang, Y.; Zhang, Y.; Ma, R.; Sun, W.; Ji, Z. Antibacterial activity of epigallocatechin gallate (EGCG) against *Shigella flexneri*. *Int. J. Environ. Res. Public Health.* **2023**, *20*, 4676, doi:https://doi.org/10.3390/ijerph20064676.

2.Obana, N.; Nakamura, K.; Nomura, N. A sporulation factor is involved in the morphological change of *Clostridium perfringens* biofilms in response to temperature. *J. Bacteriol.* **2014**, *196*, 1540–1550, doi:https://doi.org/10.1128/jb.01444-13.

3.Smith, G.L.; Reutovich, A.A.; Srivastava, A.K.; Reichard, R.E.; Welsh, C.H.; Melman, A.; Bou-Abdallah, F. Complexation of ferrous ions by ferrozine, 2,2′-bipyridine and 1,10-phenanthroline: Implication for the quantification of iron in biological systems. *J. Inorg. Biochem.* **2021**, *220*, 111460, doi:https://doi.org/10.1016/j.jinorgbio.2021.111460.

4.Ding, H.; Hu, J. The optimal method for peroxydisulfate quenching: A comparison of commonly used reductants. *Chemosphere* **2021**, *262*, 128000, doi:https://doi.org/10.1016/j.chemosphere.2020.128000.

5.Bai, M.; Li, M.; Ding, X.; Wang, Y. Quenching effect of different quenchers on $SO_4^{\bullet-}$ and $^{\bullet}OH$ based advanced oxidation processes. *Ind. Water Treat.* **2021**, *41*, 75-80, doi:https://doi.org/10.19965/j.cnki.iwt.2020-1050.

6.Wang, Y.; Zhang, M.; Kong, X.; Ma, T.; Cao, W.; Han, Z.; Wang, P.; Liu, S. Degradation of 1,2,3-trichloropropane using peroxydisulfate activation by green tea iron nanoparticles. *Environ. Technol. Innov.* **2023**, *32*, 103351, doi:https://doi.org/10.1016/j.eti.2023.103351.

7.Yuan, S.; Liao, P.; Alshawabkeh, A.N. Electrolytic manipulation of persulfate reactivity by iron electrodes for trichloroethylene degradation in groundwater. *Environ. Sci. Technol.* **2014**, *48*, 656–663, doi:https://doi.org/10.1021/es404535q.

8.Jones, C.M.; Burkitt, M.J. EPR detection of the unstable tert-butylperoxyl radical adduct of the spin trap 5,5-dimethyl-1-pyrroline N-oxide: a combined spin-trapping and continuous-flow investigation. *J. Chem. Soc., Perkin Trans. 2* **2002**, *31*, 2044–2051, doi:https://doi.org/10.1039/b207709b.

9.Niu, L.; Lin, J.; Chen, W.; Zhang, Q.; Yu, X.; Feng, M. Ferrate(VI)/periodate system: Synergistic and rapid oxidation of micropollutants via periodate/iodate-modulated Fe(IV)/Fe(V) intermediates. *Environ. Sci. Technol.* **2023**, *57*, 7051–7062, doi:https://doi.org/10.1021/acs.est.2c08965.

10.Berk, V.; Fong, J.C.N.; Dempsey, G.T.; Develioglu, O.N.; Zhuang, X.; Liphardt, J.; Yildiz, F.H.; Chu, S. Molecular architecture and assembly principles of Vibrio cholerae biofilms. *Science* **2012**, *337*, 236–239, doi:https://doi.org/10.1126/science.1222981.

11.Heydorn, A.; Nielsen, A.T.; Hentzer, M.; Sternberg, C.; Givskov, M.; Ersboll, B.K.; Molin, S. Quantification of biofilm structures by the novel computer program COMSTAT. *Microbiology* **2000**, *146*, 2395–2407, doi:https://doi.org/10.1099/00221287-146-10-2395.

12.Chang, B.; Wan, Q.; Wu, G.; Cheng, Y.; Wang, J.; Huang, T.; Wen, G. Formation of filamentous fungal biofilms in water and the transformation of resistance to chlor(am)ine disinfection. *J. Hazard. Mater.* **2024**, *476*, 135138, doi:https://doi.org/10.1016/j.jhazmat.2024.135138.

13.Ma, X.Y.; Chen, W.F.; Cheng, F.D.; Tang, L.; Wang, X.C.; Lin, Y.; L, X.K. The spectral characteristics and biotoxicity of fractions obtained from two different DOM molecular weight fractionation methods. *China Environ. Sci.* **2021**, *41*, 5885 – 5895, doi:https://doi.org/10.3969/j.issn.1000-6923.2021.12.045.
